# The lncRNA *Sweetheart* regulates compensatory cardiac hypertrophy after myocardial injury in murine males

**DOI:** 10.1038/s41467-023-42760-y

**Published:** 2023-11-02

**Authors:** Sandra Rogala, Tamer Ali, Maria-Theodora Melissari, Sandra Währisch, Peggy Schuster, Alexandre Sarre, Rebeca Cordellini Emídio, Thomas Boettger, Eva-Maria Rogg, Jaskiran Kaur, Jaya Krishnan, Gabrijela Dumbović, Stefanie Dimmeler, Samir Ounzain, Thierry Pedrazzini, Bernhard G. Herrmann, Phillip Grote

**Affiliations:** 1 Institute of Cardiovascular Regeneration, Centre for Molecular Medicine, Goethe University, Theodor-Stern-Kai 7, 60590 Frankfurt am Main, Germany; 2 Georg-Speyer-Haus, Institute for Tumor Biology and Experimental Therapy, Paul-Ehrlich-Str. 42-44, 60596 Frankfurt am Main, Germany; 3 Faculty of Science, Benha University, Benha, 13518 Egypt; 4 Department of Developmental Genetics, Max Planck Institute for Molecular Genetics, Ihnestr. 63-73, 14195 Berlin, Germany; 5 Cardiovascular Assessment Facility, University of Lausanne Medical School, Lausanne, Switzerland; 6 Department of Cardiac Development and Remodelling, Max Planck Institute for Heart- and Lung Research, 61231 Bad Nauheim, Germany; 7 Experimental Cardiology Unit, Department of Cardiovascular Medicine, University of Lausanne Medical School, Lausanne, Switzerland; 8 HAYA Therapeutics, Rte de la Corniche 6, 1066 Lausanne, Switzerland; 9 Frankfurt Cancer Institute, Goethe University Frankfurt, Frankfurt am Main, Germany

**Keywords:** Long non-coding RNAs, Cardiac hypertrophy, Cardiovascular biology

## Abstract

After myocardial infarction in the adult heart the remaining, non-infarcted tissue adapts to compensate the loss of functional tissue. This adaptation requires changes in gene expression networks, which are mostly controlled by transcription regulating proteins. Long non-coding transcripts (lncRNAs) are taking part in fine-tuning such gene programs. We describe and characterize the cardiomyocyte specific lncRNA *Sweetheart RNA* (*Swhtr*), an approximately 10 kb long transcript divergently expressed from the cardiac core transcription factor coding gene *Nkx2-5*. We show that *Swhtr* is dispensable for normal heart development and function but becomes essential for the tissue adaptation process after myocardial infarction in murine males. Re-expressing *Swhtr* from an exogenous locus rescues the *Swhtr null* phenotype. Genes that depend on *Swhtr* after cardiac stress are significantly occupied and therefore most likely regulated by NKX2-5. The *Swhtr* transcript interacts with NKX2-5 and disperses upon hypoxic stress in cardiomyocytes, indicating an auxiliary role of *Swhtr* for NKX2-5 function in tissue adaptation after myocardial injury.

## Abstract (web summary)

LncRNAs take part in fine-tuning gene regulatory networks in development, homeostasis, and disease settings. Here, the authors show that the lncRNA Sweetheart has an important role in cardiomyocytes after myocardial injury to act together with NKX2-5 in adapting gene programs after myocardial stress.

## Introduction

Precise regulation of gene expression networks is required to form a healthy heart and to maintain proper heart function after birth and throughout adulthood. Such networks not only contain protein-coding genes but also many long non-coding genes (IncRNAs), which are as abundant as coding genes^1,2^. The function and mechanism of these IncRNAs vary greatly, but they are often associated with transcriptional regulation^3^. Two major types of mechanisms are typically discussed when referring to IncRNA gene function. For some loci, the resulting RNA is just a circumstantial byproduct with the act of transcription being the major bearer of its function. One such example in the cardiac system is the lncRNA locus *Handsdown RNA* (*Hdnr*), downstream of the *Hand2* transcription factor coding gene. During embryonic development, modification of the *Hdnr* transcriptional activity alters the *Hand2* expression levels, but not modifying the RNA levels of *Hdnr*^4^. One example of IncRNA loci that exhibit an RNA-based mechanism is the cardiac-specific *Myosin Heavy Chain Associated RNA Transcripts* (*MyHEART*; *Mhrt*), a cluster of IncRNAs that undergo anti-sense transcription from the *myosin heavy chain 7* (*Myh7*) locus. Transaortic constriction (TAC) induced pathological stress results in *Mhrt* downregulation by *Brg1* upregulation and subsequent BRG1-mediated chromatin remodeling in vivo. Thus, hypertrophy-related gene programs are initialized. As the binding of *Mhrt* to BRG1 antagonizes its DNA binding capability, preserving *Mhrt* expression levels after TAC prevents cardiac hypertrophy and heart failure^5^.

One of the core regulators of heart development is the cardiac-specific homeobox protein NKX2-5, which is present in early cardiomyocytes already at embryonic day (E) 7.5 during murine embryonic development. Systemic deletion of the *Nkx2-5* gene in mice causes defects in heart looping and the formation of ventricular structures. In addition, other important cardiac regulatory genes are dysregulated and as a combined result the embryos exhibit early embryonic lethality^6^. *Nkx2-5* is not silenced after birth and is abundantly expressed in adult heart tissue^7^. However, not much is known about its function in terminally differentiated cardiac tissue and its involvement in cardiac maintenance and disease. In human patients suffering from congenital heart disease, *Nkx2-5* mutations are commonly found^8^, however, the precise involvement of *Nkx2-5* in the disease context in adult patients remains unknown.

Heart disease represents the main cause of death in the developed world; acute myocardial infarctions (AMI) being the most common form. Reduced blood flow leads to decreased oxygen supply of the heart tissue and thus irreversible damage, such as apoptosis and necrosis of cardiomyocytes and formation of scar tissue which results in a loss of flexibility. Due to the limited regenerative capacity of the terminally differentiated heart tissue, the remaining viable tissue adapts through other mechanisms, such as hypertrophic remodeling that involves the thickening of the ventricular walls by an increase of the cardiomyocyte cell size^9^. While cardiac hypertrophy in response to pathological stimuli is often associated with heart failure, we show that it is necessary for survivability after cardiac injury in mice.

Here, we characterize a cardiac-specific IncRNA, which we termed *Sweetheart RNA* (*Swhtr*) that is required for the regulation of hypertrophic gene programs, most likely acting in concert with NKX2-5.

## Results

### *Swhtr* is a nuclear lncRNA specifically expressed in the heart

**Fig. 1:**
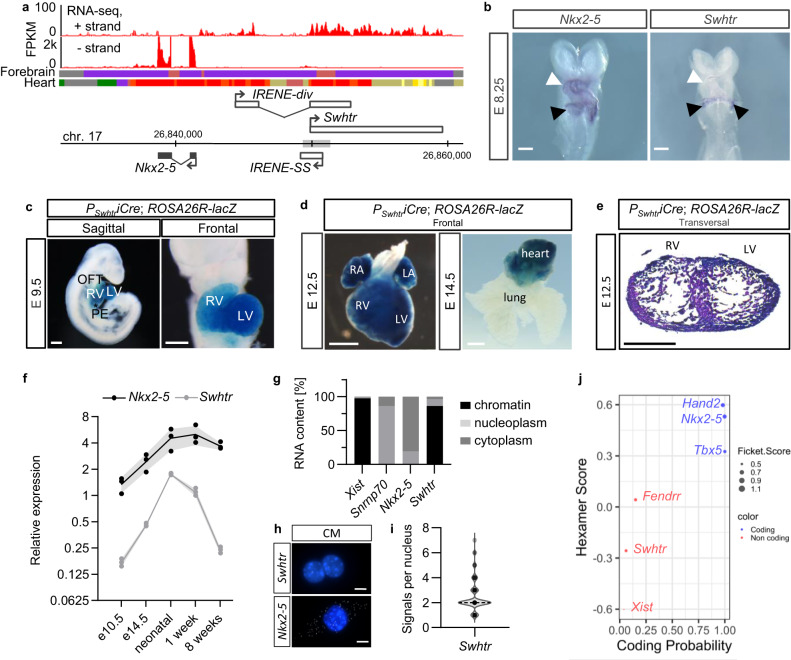
Expression and localization of the *Sweetheart* lncRNA. **a** Strand-specific RNA-seq from E9.5 heart tubes showing the *Nkx2-5* region. (gray box = first heart field enhancer). Plus-strand track is 20× amplified over the minus-strand track. The chromatin state tracks are given for the forebrain and heart from ENCODE in ChromHMM colors (red = TSS, yellow/orange = enhancers, light gray = poised enhancer, purple = heterochromatin, dark gray = quiescent). The number above the genome bar denotes the *mm10* coordinates and the vertical tip bars represent 10,000 bp steps. The coordinates for the Swhtr transcription unit are chr17:26,849,907–26,859,715 (mm10). **b** Whole mount in situ hybridization of *Nkx2-5* and *Swhtr* in E8.25 embryos. White arrows show the early heart tube with two different heart fields and black arrowhead points to the inflow tract region. The white line represents 500 µm and the staining was repeated two times. **c** Lineage tracing of *Swhtr* expressing cells in E9.5 embryos. OFT outflow tract, RV right ventricle, LV left ventricle, PE pre-pericardium. The white line represents 500 µm. **d** Lineage tracing of *Swhtr* expressing cells in late gestation embryos and heart/lung explant. The white line represents 1 mm. **e** Lineage tracing of *Swhtr* expressing cells in a transversal section of an E12.5 heart. RV right ventricle, LV left ventricle. The black line represents 500 µm. **c**–**e** are representative images from one of two independent transgenic lines that showed the same staining pattern **f** Quantitative Real-Time PCR timeline of *Swhtr* and *Nkx2-5* expression levels in the hearts of E10.5 embryos to 8-week adult mice. Embryo hearts were pooled from independent litters and the postnatal stages represent data from individual hearts (*n* = 3). Error bars are given as SEM. **g** Subcellular fractionation of E11.5 cardiomyocytes (CMs) of marker transcripts and *Swhtr* (*n* = 2). **h** SmFISH of *Nkx2-5* and *Swhtr* in 24 h cultured neonatal cardiomyocytes. The white line represents 5 µm. **i** Quantification of smFISH *Swhtr* signal in 24 h cultured neonatal cardiomyocytes. Note that on average two dots were found in the nucleus (*n* = 86). **j** Analysis of coding potential of *Swhtr* by CPAT compared to known coding and non-coding RNAs.

In a previously generated dataset that identified the transcriptional landscape of different tissues of early mid-gestation mouse embryos^10^ we identified an RNA that is expressed exclusively in heart tissue. This RNA, which we termed *Sweetheart RNA* (*Swhtr*), is divergently expressed from the essential, transcription factor coding gene *Nkx2-5*^6^. Its annotation partially overlaps with the previously described IncRNA *IRENE-div*^11^. We determined its major transcript by 5’ and 3’ RACE PCR and found that the major variant from *Swhtr* locus is 9809 nucleotides in length and bears no introns (Fig. 1a). The transcriptional start site (TSS) maps to a previously described GATA4 bound first heart field specific enhancer located ~8 kb upstream of *Nkx2-5*^12^. To characterize whether *Swhtr* is specific for the first heart field we conducted whole mount in situ hybridization (WISH) in E8.25 mouse embryos and found that whereas *Nkx2-5* is expressed in the whole heart tube at that stage, *Swhtr* expression is restricted to the early inflow tract of the developing heart (Fig. 1b). Lineage tracing experiments of *Swhtr* expressing cells confirmed that while *Swhtr* expressing cells contribute to both ventricles, the left loop that originates from the first heart field exhibits a much more even staining (Fig. 1c). The right loop that originates mostly from the second heart field exhibits a more salt-and-pepper like staining (Fig. 1c), like already in the in situ staining (Fig. 1b). In heart and lung of later stage embryos (E12.5; E14.5) the staining within the left and right ventricle is even with no traces of *Swhtr* expressing cells or their descendants found in neither lung nor epicardial tissue (Fig. 1d, e). Compared to the *cis* located *Nkx2-5* gene, *Swhtr* is much lower expressed (Fig. 1a, f). Expression analysis from the whole heart at different stages shows that expression levels of *Nkx2-5* and *Swhtr* are changing comparably (Fig. 1f).

To investigate where the *Swhtr* transcript localizes intracellularly, we conducted subcellular fractionation of embryonic cardiomyocytes. Compared to marker transcripts known to be localized to the chromatin, nucleoplasm and cytoplasm fraction, the *Swhtr* IncRNA localizes predominantly to the chromatin fraction within the nucleus (Fig. 1g). We validated these findings by single-molecule fluorescence in situ hybridization (smFlSH) experiments that revealed two distinct fluorescent *Swhtr* signals within the nucleus, suggesting that *Swhtr* might reside at its site of transcription (Fig. 1h, i). Presence of the full-length transcript was validated by antisense-oligo (ASO)-mediated knockdown targeting the 3’-end and subsequent verification of depletion by 5’ directed qRT-PCR primers (Supplementary Fig. 1). It has become clear, that some RNAs that are classified as lncRNAs can encode micro peptides, which might be functional. However, these are usually cytoplasmic localized^13^ pointing against a functional open-reading frame (ORF) contained in *Swhtr*. CPAT analysis^14^ further demonstrates that *Swhtr* has very low coding potential (Fig. 1j). In conjunction with the localization data this points towards a purely non-coding transcript. Together this data shows that *Swhtr* is a chromatin-bound IncRNA, specifically expressed in the heart of developing mice and at postnatal stages.

### Genetic inactivation of *Swhtr* does not affect heart development and homeostasis

**Fig. 2:**
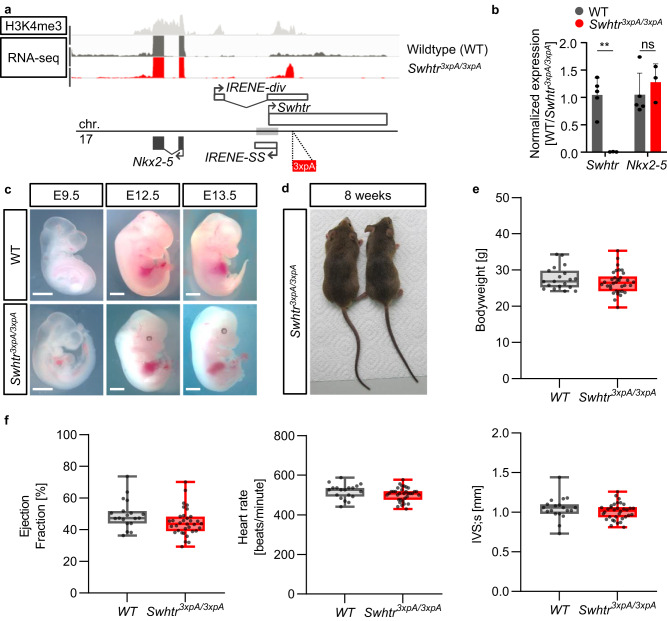
No overt phenotype in *Swhtr*^*3xpA/3xpA*^ mutant embryos or adult mice. **a** ChIP-seq (H3K4me3) and RNA-seq (E9.5 heart tubes) from WT and *Swhtr*^*3xpA/3xpA*^ mutant mice. **b** qRT-PCR validation of loss of *Swhtr* in E9.5 embryonic hearts (WT *n* = 5; *Swhtr*^*3xpA/3xpA*^ *n* = 3). Values are given in mean ± SEM. Statistical significance was tested by Two-way ANOVA with Šídák’s multiple comparisons test. ns not significant, **0.0016. **c** Embryos of indicated age from WT and *Swhtr* mutants. The white line represents 1 mm. Images are representative of three independent litters. **d** Eight-week-old *Swhtr*^*3xpA/3xpA*^ founder mice. **e** Bodyweight of *Swhtr*^*3xpA/3xpA*^ eight times backcrossed (C57Bl6J) mice (WT *n* = 19; *Swhtr*^*3xpA/3xpA*^ *n* = 34). **f** Selected cardiac parameters determined by echocardiography in 8-week-old mice of the indicated genotype (WT *n* = 19; *Swhtr*^*3xpA/3xpA*^ *n* = 34). Statistical significance was tested by two-way ANOVA. No statistically significant differences were detected.

To investigate the physiological function of *Swhtr* we genetically engineered a knock-out mouse in which we inserted a strong transcriptional stop signal (3xpA) to abolish *Swhtr* expression. To avoid any conflicts with existing regulatory elements we inserted this stop signal downstream of a phylogenetically conserved region of the GATA4 bound enhancer. This genetic insertion causes premature termination of the transcriptional process and, hence, a severely shortened *Swhtr* transcript (Fig. 2a). The full-length *Swhtr* RNA is not detectable anymore when we profiled E9.5 hearts by either RNA-seq or qPCR (Fig. 2a, b), demonstrating that the *Swhtr* transcriptional start site (TSS) locates upstream of the inserted transcriptional stop signal and no other alternative transcript is initiated from any downstream elements residing in the *Swhtr* transcription unit. While the *Swhtr* RNA is lost, the expression level of its *cis* located gene *Nkx2-5* in the heart is unchanged at that stage and under these conditions (Fig. 2b). In addition, the expression levels of the other described lncRNAs, *IRENE-SS* and *IRENE-div* are not affected in our knock-out (Supplementary Fig. 2a). Furthermore, acute depletion of *Swhtr* RNA in HL-1 cardiomyocytes by ASO knockdown does not influence *Nkx2-5* transcript levels, also arguing against a role of *Swhtr* in regulating its *cis* located gene *Nkx2-5* on the transcriptional level (Supplementary Fig. 1). While *Swhtr* might be an alternative transcript from the same locus as *IRENE-div*, this finding sets *Swhtr* apart from the *Nkx2-5* regulating *IRENE-div* eRNA and provides evidence for an independent function of *Swhtr*.

Phenotypically, homozygous *Swhtr null* embryos do not display differences compared to wild-type embryos during development and grow up into adult animals with no overt defects (Fig. 2c, d). To identify subtle phenotypic changes in adult hearts as a result of *Swhtr* lacking throughout development, we investigated the heart function in *Swhtr null* animals by echocardiography after backcrossing the *Swhtr null* mutants to the C57BL/6J genetic background. We compared their body weight and cardiac parameters, such as ejection fraction, heart rate and left ventricular diameter of adult *Swhtr null* and wild type mice. Neither the body weight nor any of the heart parameters differed significantly from that of wild-type mice (Fig. 2e, f and Supplementary Fig. 2b). We conclude that under standard breeding conditions, *Swhtr* is dispensable for normal heart function.

### *Swhtr* is required for a compensatory response of the heart after myocardial infarction

**Fig. 3:**
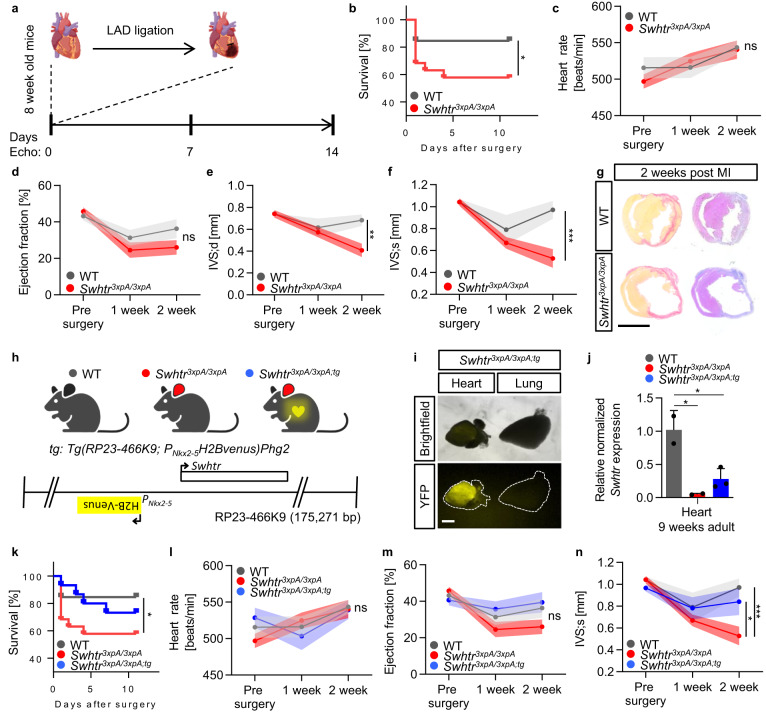
Induced myocardial infarction by left ascending artery ligation (LAD). **a** Schematic of the analysis setup for echocardiography and LAD ligation in mice of age 8 weeks. The schematic of the hearts was taken from BioRender.com. **b** Reduced survival of *Swhtr null* mice compared to WT mice after LAD ligation (WT *n* = 13; *Swhtr*^*3xpA/3xpA*^ *n* = 19). Statistical significance was tested by Kaplan–Meier simple survival analysis. *0.0387. **c**–**f** Selected heart-specific parameters in mice (*n* = 9 animals per genotype) before and after (1 and 2 weeks) LAD ligation. Values are given in mean ± SEM. Statistical significance was tested by Two-way ANOVA with Tukey correction for multiple comparisons. ns not significant, **0.0012, ***0.0002. **g** Verification of infarct presence in mice 2 weeks after LAD ligation by Sirius red (fibrotic tissue) staining. The black line represents 5 mm. Images are representative of three independent heart sections. **h** Schematic of the rescue transgene and the resulting mouse line. The rescue transgene (*tg*) is comprised of the BAC (RP23-466K9) that includes the *Nkx2-5* (H2Bvenus inserted in *Nkx2-5* ATG) and *Swhtr* loci, randomly inserted into the genome of wild type C57BL6J mice. The color key for the genotype was generated using BioRender.com. **i** Verification of *tg* presence and activity by heart-specific presence of H2BVENUS. Image shows organs prepared from one specimen from the first litter **j** Expression verification of *Swhtr* in from the *tg* in *Swhtr null* mutants, after crossing (WT *n* = 2; *Swhtr*^*3xpA/3xpA*^ *n* = 2; *Swhtr*^*3xpA/3xpA;tg*^ *n* = 3). Values are given in mean ± SEM. Statistical significance was tested by One-Way ANOVA with Tukey correction for multiple comparisons. WT/*Swhtr*^*3xpA/3xpA*^ *p* = 0.0136, WT/*Swhtr*^*3xpA/3xpA;tg*^ *p* = 0.025. **k** Reduced survival of *Swhtr null* mice compared to WT and *Swhtr* rescue mice after LAD ligation (WT *n* = 13; *Swhtr*^*3xpA/3xpA*^ *n* = 19; *Swhtr*^*3xpA/3xpA;tg*^ *n* = 15). Statistical significance was tested by Kaplan–Meier Simple Survival Analysis. *0.0387. **l**–**n** Selected heart-specific parameters in mice (*n* = 9 animals per genotype) before and after (1 and 2 weeks) LAD ligation. Values are given in mean ± SEM. Statistical significance was tested by Two-way ANOVA with Tukey correction for multiple comparisons. ns = not significant, *0.0248, ***0.0002.

Many IncRNA knock-out animal models do not display an overt phenotype after genetic depletion under standard conditions^15–17^. One possibility is that these analyzed lncRNA genes are actually not functional^18^. Another possibility is that a functional requirement is only detected under stress conditions. To challenge the heart we selected the left anterior descending artery (LAD) ligation model, which induces a myocardial infarction in the lateral left ventricle^19^. An acute myocardial infarction (AMI) was induced in male mice of 8 weeks of age and heart parameters were monitored by echocardiography pre-infarction and at day 7 and day 14 after the infarction (Fig. 3a). After 14 days animals were sacrificed, and the presence of infarct tissue was verified by Sirius red staining (Fig. 3g). Notably, compared to wild type mice, *Swhtr null* animals displayed increased mortality after AMI (Fig. 3b), while the size of the myocardial infarction (MI) was similar between the groups (Supplementary Fig. 3). This indicates that albeit no difference is observed in our measured heart parameters, an early response to the AMI already requires *Swhtr*. In the surviving animals, most parameters of cardiac function did not change significantly, but strikingly, the interventricular septum (IVS) of *Swhtr null* mice did not display compensatory thickening after AMI compared to wild-type (Fig. 3c–g and Supplementary Fig. 3). This establishes that *Swhtr* is involved in the early stress response after AMI and in the adaptive response of the cardiac tissue after myocardial infarction.

To determine whether the *Swhtr-*dependent adaptation after AMI is a result of the loss of the RNA transcript or the loss of the transcriptional activity at this locus, we generated a *Swhtr* rescue mouse line that re-expresses the *Swhtr* IncRNA from an exogenous locus (random single-copy BAC insertion) (*Tg(RP23-466K9; P*_*Nkx2-5*_*H2Bvenus)Phg2*) (Fig. 3h). This rescue construct contains an *H2BVenus* fusion expression cassette instead of the *Nkx2-5* coding sequence to detect activity of the transgene and to avoid having an additional third copy of the *Nkx2-5* locus present in our genetic setup. Consequently, mice from this transgenic line show yellow fluorescence exclusively in the heart (Fig. 3i) and re-express *Swhtr* (Fig. 3j). We crossed this wild-type mouse line to our *Swhtr null* mice to generate the *Swhtr* rescue line (*Swhtr*^*3xpA/3xpA; tg*^).

The *Swhtr* rescue mice resembled the wild-type control mice in regard to mortality (Fig. 3k) and the size of the IVS (Fig. 3n) after AMI, while neither of the remaining parameters analyzed show significant differences (Fig. 3l, m and Supplementary Fig. 3). The lack of a functional *Nkx2-5* gene next to the *Swhtr* locus in our rescue transgene indicates that *Swhtr* function does not require the presence of *Nkx2-5* in *cis*, hence, a role of *Swhtr* in regulating *Nkx2-5* expression in this setting can be excluded. We also conclude that the *Swhtr* transcript is important for its function.

### *Swhtr* is required for hypertrophic re-modeling after myocardial infarction

**Fig. 4:**
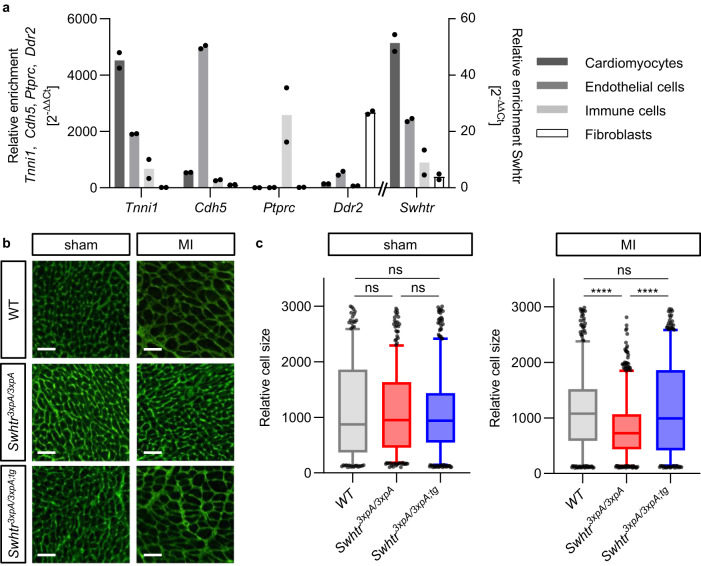
Hypertrophy in *Swhtr* mutant cardiomyocytes. **a** Relative enrichment of *Swhtr* in the main cell-types represented in the heart of 8-week-old mice as compared to marker genes (*n* = 2). Note that the *Swhtr* expression pattern resembles that of *Tnni1*. **b** Wheat Germ Agglutinin stained IHC of representative sections of IVS tissue in the indicated genotypes 2 weeks post sham or MI. The white line represents 50 µm. **c** Automated quantifications of relative cell sizes in the interventricular septum of three representative animals of the indicated genotype 2 weeks post sham or MI. Plotted are 5–95 percentile and the middle line of the box plot represents the median. Statistical significance was tested by One-way ANOVA with Dunnett correction for multiple comparisons. ns not significant, ****p < 0.0001.

The thickening of the interventricular septum after the induced myocardial injury in wild-type and *Swhtr* rescue mice might be a result of a hypertrophic response of the remaining muscle tissue. To address whether this might be due to an inherent function of *Swhtr* in cardiomyocytes we first determined the expression of *Swhtr* in different heart cell populations^20^. We found upon fractionation of the four major cell types in the adult heart (8 weeks) that *Swhtr* exhibits the same pattern as the cardiomyocyte marker gene *Tnni1*, indicating cardiomyocyte specificity of *Swhtr* in adult hearts (Fig. 4a). Then, we investigated the presence of larger cells, reminiscent for hypertrophy, in sections of hearts from the LAD-Iigation experiment. Wheat Germ Agglutinin (WGA) staining revealed that the IVS region of wild type and *Swhtr* rescue indeed exhibit on average increased cell size (Fig. 4b,c). In contrast, the cell size in the *Swhtr null* IVS even decreased significantly (Fig. 4c). This establishes that the *Swhtr* RNA is required for the adaptive hypertrophic response of the cardiomyocyte tissue after a myocardial infarction.

### *Swhtr* regulates NKX2-5-mediated cardiac stress response

**Fig. 5:**
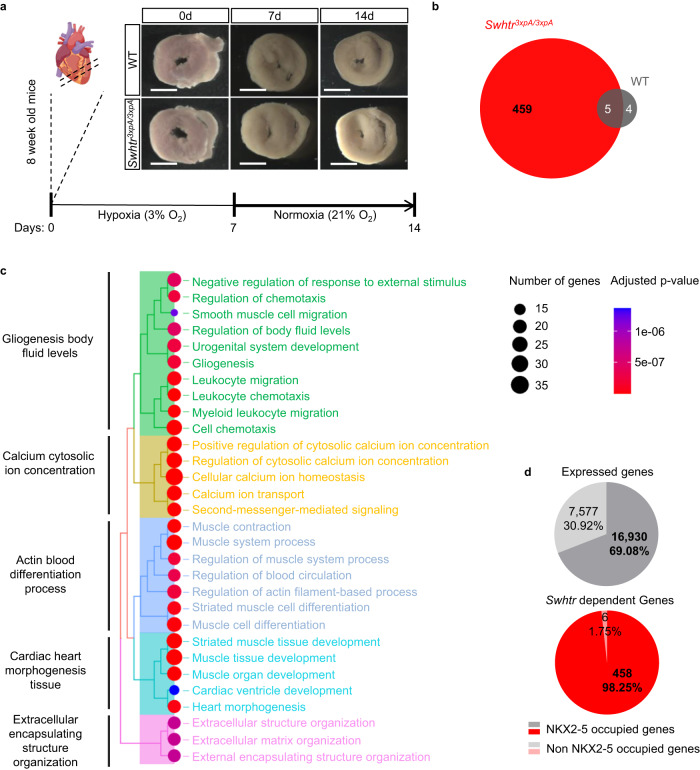
*Swhtr* dependent genes under cardiac stress. **a** Schematic overview of the experimental procedure with representative pictures of heart slices (slices derived from 4 male mice per genotype from two independent litters). The white line represents 5 mm. The schematic of the heart was taken from biorender.com. **b** Number of deregulated genes after 7 days of hypoxia treatment followed by 7 days of normoxic conditions of *Swhtr*^*3xpA/3xpA*^ and WT slices compared to WT heart slices (*n* = 4). **c** GO-term enrichment analysis of deregulated *Swhtr-dependent* genes. **d** NKX2-5 occupation on expressed genes that are not dysregulated (gray) and on the 464 *Swhtr-dependent* genes (red). Percental distribution was tested for significant differences between *Swhtr-dependent* and expressed genes by binomial test (*p* < 0.0001).

Scar formation is one of the consequences of myocardial infarction (Fig. 3e). The resulting heterogeneity of the infarcted heart tissue can lead to biased RNA profiling. To mitigate this compositional bias and obtain consistent RNA profiling we performed primary tissue culture of defined heart tissue slices. Replicate slices were either cultivated continuously for 2 weeks under normoxic condition (untreated) or 1 week under hypoxic condition, followed by 1 week under normoxic condition (treated), mimicking our in vivo AMI stress model (Fig. 5a). When we compared expression profiles of wild type treated versus untreated heart slices, we found only 9 genes to be dysregulated (Fig. 5b). In contrast, 464 genes were dysregulated in *Swhtr null* heart slices when profiles of treated were compared to untreated tissue (Fig. 5b). The near absence of dysregulated genes in wild type compared to the high rate of dysregulated genes in mutant heart slices indicates that *Swhtr* is required for recovery after cardiac stress (Fig. 5b). GO-term analysis shows that these *Swhtr* dependent genes are mainly involved in biological processes such as muscle function, heart morphogenesis and extracellular matrix organization, leukocyte migration and chemotaxis indicative of inflammatory response, intracellular calcium homeostasis, all of which are integral components of cardiac stress response (Fig. 5c). Accordingly, KEGG pathway analysis reveals pathways involved in inflammatory response, cardiomyopathy, glucose metabolism and response to oxygen levels depend on *Swhtr* (Supplementary Fig. 4a). The loss of *Swhtr* does not lead to changes in *Nkx2*-*5* expression level neither in adult hearts (Fig. 2b, Supplementary Fig. 2a) nor in our treated heart slice culture (Fig. 5b), the localization of *Swhtr* to its locus of transcription (Fig. 1i) implicates some involvement of *Nkx2-5* in this process. To determine if a significant number of *Swhtr-*dependent genes are occupied by NKX2-5 and therefore might be direct targets we analyzed available ChIP-seq data of NKX2-5 occupation in adult heart tissue^21^. We found that almost all 464 dysregulated genes (98.27%) are occupied by NKX2-5 (Fig. 5d, Supplementary Fig. 4b), while only 69.08% of all genes that are expressed in our heart slices without the 464 dysregulated genes are occupied by NKX2-5. This indicates that the lack of *Swhtr* impairs NKX2-5-mediated response to cardiac stress, suggesting that *Swhtr* might act together with NKX2-5 to regulate this cardiac stress response.

### *Swhtr* interacts with NKX2-5 and disperses from its locus under hypoxic stress

**Fig. 6:**
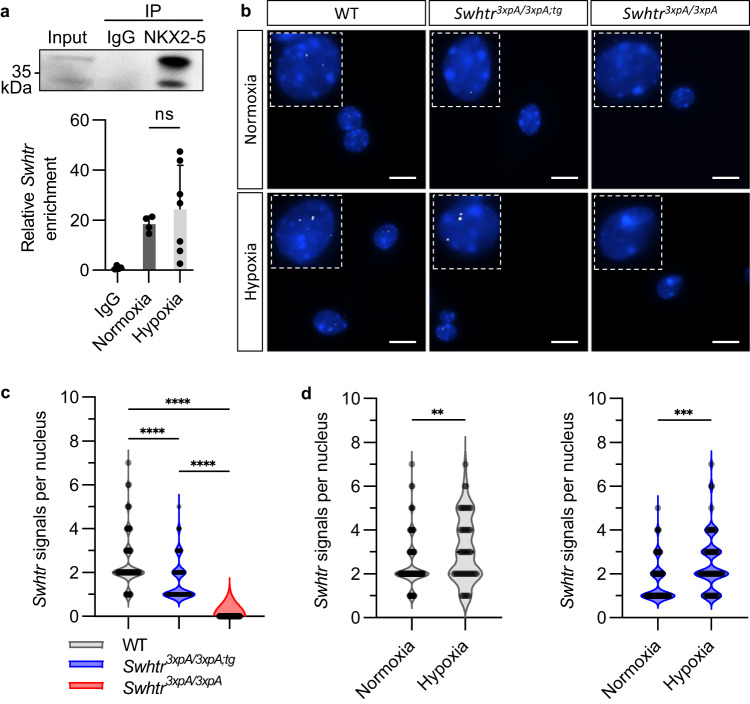
*Swhtr* dispersion under hypoxic stress. **a** RNA immunoprecipitation of NKX2-5. Western Blot detection of NKX2-5 enrichment over input after pulldown. qRT-PCR analysis of relative enrichment of *Swhtr* in NKX2-5 pulldown of HL-1 cardiomyocytes under normoxic (21% O_2_) and hypoxic (1% O_2_) conditions (24 h) normalized to IgG negative control (IgG *n* = 4, Normoxia *n* = 4, Hypoxia *n* = 7). Values are given in mean ± SEM. **b** smFISH of *Swhtr* under normoxic (21% O_2_) and hypoxic (1% O_2_) conditions (24 h) in WT, *Swhtr*^*3xpA/3xpA;tg*^ and *Swhtr*^*3xpA/3xpA*^ neonatal cardiomyocytes. The white line represents 10 µm. **c** Quantification of smFISH *Swhtr* signals in the nucleus counted from isolated neonatal cardiomyocytes of 4 animals of the indicated genotype (*n* > 50). Color legend applies to (**c**) and (**d**). **d** Quantification of smFISH *Swhtr* signals in the nucleus of neonatal cardiomyocytes isolated from 4 WT or *Swhtr*^*3xpA/3xpA;tg*^ animals under normoxic (21% O_2_) and hypoxic (1% O_2_, 24 h) conditions (*n* > 50). Statistical significance was tested by Two-way ANOVA with Tukey correction for multiple comparisons. **0.0031, ***0.0006, ****<0.0001.

As all our data do not indicate a function of *Swhtr* on *Nkx2-5* expression, the other possibility is that *Swhtr* could act together with NKX2-5 by direct interaction. It was shown previously that *IRENE-SS*, but not *IRENE-div* can interact with NKX2-5^11^. We found that when we pull-down NKX2-5 from HL-1 cardiomyocytes *Swhtr* transcript co-precipitates (Fig. 6a). This interaction, though highly variable, seems to increase under hypoxic conditions. To explore how *Swhtr* is able to regulate target genes together with NKX2-5, although it resides mostly at its locus of transcription (Fig. 1h, i), we performed smFISH analysis on isolated P0 cardiomyocytes from either wild type, *Swhtr null* or our rescue mouse line under normoxic and hypoxic conditions. We detected an average of two signals in wild-type cardiomyocytes and one signal in cardiomyocytes from the *rescue* mouse line, correlating with the number of active *Swhtr* transcription sites (Fig. 6b, c). Strikingly, under hypoxic conditions, we observe that the *Swhtr* transcript disperses from its locus of origin, as we detect multiple signals in single nuclei of wild type as well as rescue cardiomyocytes (Fig. 6b, d). We find the same dispersion of *Swhtr* in HL-1 cardiomyocytes (Supplementary Fig. 5). The data show that under stress conditions *Swhtr* transcripts, possibly bound to NKX2-5, disperse from their locus of origin. The combined data suggest that *Swhtr* bound to NKX2-5 together regulate particular target genes under these conditions. Moreover, the data reveal how a lncRNA can act in *trans* at multiple loci under stress conditions, although under normal conditions it resides at its locus of origin.

## Discussion

Here we characterize an IncRNA transcript that we termed *Swhtr*, which partially overlaps with a previously published lncRNA from the same genomic region: *IRENE-div*^11^. In contrast to these previously described eRNAs, our study shows that the absence of the long *Swhtr* transcript does not lead to persistent or immediate *Nkx2-5* mRNA dysregulation, neither during embryonic development nor during cardiac homeostasis. Hence, although *Swhtr* might be an isoform from the same locus as *IRENE-div*, it has an independent function. We show by fractionation and smFISH that the *Swhtr* lncRNA is localized to chromatin and stays mostly at its locus of origin. Together with the high non-coding potential, this argues that no embedded micropeptide within the nearly 10 kb long *Swhtr* transcript is involved in its function. Our data clearly shows an RNA-based function for *Swhtr*, as re-expression of *Swhtr* from an exogenous locus in *Swhtr null* mutants rescues the lethality after AMI and the defect in developing cardiac hypertrophy. The promoter of *Swhtr* was described previously as an *Nkx2-5* cardiac enhancer element active in multipotent cardiac progenitor cells^12,22^. The *Swhtr* promoter becomes active after myocardial stress, which was described previously^23^. These multipotent cardiac progenitor cells were injected into infarcted hearts, but it seems they cannot contribute to the regeneration of the heart tissue. Our data rather indicate that this genetic element is important to activate *Swhtr* and that this activity is required for an early response after a stress stimulus and the downstream tissue remodeling of the remaining intact cardiac tissue after infarction.

The *cis* located gene to *Swhtr* is the core cardiogenic transcription factor coding gene *Nkx2-5. Nkx2-5* is known to be required for embryonic development of the cardiac system^6^. However, although the locus is still active and abundantly expressed in adults, not much is known about its function after birth and in cardiac stress. Interfering with NKX2-5 function by over-expression of a dominant negative *Nkx2-5* mutant has been shown to lead to apoptosis of cultured cardiomyocytes. In contrast, over-expression of a wild-type form of *Nkx2-5* has a protective effect against doxorubicin-induced stress^24^. This demonstrates the critical role of *Nkx2-5* in the maintenance of adult cardiomyocytes as well as in the response to stress. Here, we show significant NKX2-5 occupation on *Swhtr-dependent* genes. Moreover, we show here that the *Swhtr* transcript can interact directly with the NKX2-5 transcription factor. We further show that the *Swhtr* transcript disperses from its locus of origin under immediate hypoxic stress after just 24 h, indicating that this early reaction involving *Swhtr* might be important for an early response and partially protects against lethality after severe stress like AMI. The surviving animals of this immediate stress fail to develop hypertrophy in the remaining cardiac tissue. However, the *Swhtr* transcript disperses under an immediate stress stimulus already after 24 h, indicates that *Swhtr* is involved in early events after AMI. Moreover, *Swhtr* seems to act in concert with NKX2-5 under these immediate stress conditions and initiate cellular changes that lead to the initiation of hypertrophy-associated gene programs later on. By overexpression of human *NKX2-5* in adult mice, it was shown that while normal morphogenesis and function are not altered in these hearts, typical NKX2-5 target genes are dysregulated. In contrast, overexpression of *NKX2-5* in skeletal muscle, where *Swhtr* is not expressed, does not lead to the same dysregulation of NKX2-5 targets, suggesting that an additional co-factor is needed for NKX2-5 in the heart^25^. Furthermore, mutations in *NKX2-5* have been associated previously with dilated cardiomyopathy^26^. We show that *Nkx2-5* and its wider locus is an important responder to cardiac stress in adult hearts in vivo, acting in concert with its divergently expressed lncRNA *Swhtr*.

In response to increased demands on the remaining tissue after injury, the viable tissue adapts by cardiac hypertrophy to maintain blood supply to the body^9^. This is demonstrated by the increase of average cell size in the IVS of wild-type animals after AMI, together with the heart parameters being unaffected. Our *Swhtr null* mice have defects in this hypertrophic response after cardiac injury. While the differences in ejection fraction do not reach statistical significance, the observed decrease meets the requirements of a trend in loss of heart functionality. Together with the increase of lethality after immediate myocardial injury, this points towards a cardioprotective role of the *Swhtr* lncRNA by making the cardiac tissue permissive for a hypertrophic response after myocardial injury. This is further supported by our ex vivo analysis of heart slices. One of the main pathways known to be involved in the hypertrophic process is the phosphoinositide 3-kinase (PI3-K) pathway that is, among others, responsible for metabolic substrate utilization and function of cardiomyocytes^27^. The RNA profiling of cardiac slices subjected to hypoxic stress and following recovery time revealed dysregulation of 464 genes in slices derived from *Swhtr null* animals as compared to only 9 dysregulated genes in wild-type slices. Notably, KEGG pathway enrichment analysis revealed that AGE-RAGE signaling is among the most affected pathways. Even though it is not frequently discussed in relation to cardiac hypertrophy, it is a pathway known to be involved in the stress response of cardiomyocytes to stimuli such as oxidative stress^28,29^. Additionally, AGE-RAGE signaling is known to be an inducer of the PI3-K pathway, coherent with its role in hypertrophic remodeling.

Maladaptive hypertrophy, in contrast, is defined as occurring after pathological stimuli and is associated with heart failure and disease^30^. Among others, inflammatory response, calcium homeostasis and signaling, and extracellular matrix deposition are biological processes involved in maladaptive cardiac hypertrophy^31^. These biological processes are significantly impaired upon loss of *Swhtr*, indicative of maladaptive behavior. It has been discussed that an initial adaptive hypertrophic response can transition to maladaptive hypertrophy upon persistent pathological stress^31^. Within the scope of the experiment, no decrease in heart function could be detected in animals capable of hypertrophic remodeling, despite the deregulation of maladaptive hypertrophic processes in dependence of *Swhtr*. Additionally, the increased survival of wild-type animals compared to *Swhtr* mutant animals suggests early processes after myocardial stress are already important for hypertrophic remodeling to occur.

One limitation of this study is how the early response after AMI and the development of hypertrophy are connected. It will be interesting to observe transcriptional changes in discreet cell types right after an AMI event and track these adaptive changes to the development of hypertrophy in the surviving cohort. An overlay of such transcriptome changes in connection with NKX2-5 occupation in wild-type and *Swhtr null* mice at early and later stages after the AMI event might reveal the direct target genes and enable the determination of binding modalities of NKX2-5 that depend on the *Swhtr* transcript.

Even though the detailed mechanism remains to be determined we here show a clear cardioprotective role of the murine lncRNA *Swhtr* and a compensatory gene regulatory network depending on *Swhtr*. While no lncRNA was described yet for the human *NKX2-5* locus, the promoter element of *Swhtr* is highly conserved across placental species. When we analyzed publicly available RNA-seq data from human iPSCs and cardiomyocytes derived from such iPSCs^32^, we detected transcripts upstream of *NKX2-5* and, hence, orthologue transcripts for *IRENE-SS* (*ENSG00000289493*), *IRENE-div* and *SWHTR* (Supplementary Fig. 6). While the sequences are not conserved, the genetic element contributing to their expression is. Further investigation will show whether this *SWHTR* locus might have the same cardioprotective role in humans and if this function could be employed for therapeutic application.

## Methods

### Culturing of mouse ES cells

The genetic background of the ES cells generated in this work is identical (129S6/C57BL6 (G4))^33^ or C57BL/6J (gift from Lars Wittler). The mESCs were either cultured in feeder-free 2i media or on feeder cells (mitomycin-inactivated SWISS embryonic fibroblasts) containing LIF1 (1000 U/ml). 2i media: 1:1 Neurobasal (Gibco #21103049):F12/DMEM (Gibco #12634-010), 2 mM l-glutamine (Gibco), 1x Penicillin/Streptomycin (100x penicillin (5000 U/ml)/streptomycin (5000 μg/ml), Sigma #P4458-100ML, 2 mM glutamine (100x GlutaMAX™ Supplement, Gibco #35050-038), 1x non-essential amino acids (100x MEM NEAA, Gibco #11140-035), 1x Sodium pyruvate (100x, Gibco, #11360-039), 0.5x B-27 supplement, serum-free (Gibco # 17504-044), 0.5x N-2 supplement (Gibco # 17502-048), Glycogen synthase kinase 3 Inhibitor (GSK-Inhibitor, Sigma, # SML1046-25MG), MAP-Kinase Inhibitor (MEK-Inhibitor Sigma, #PZ0162), 1000 U/ml Murine_Leukemia_Inhibitory_Factor ESGRO (10^7^ LIF, Chemicon #ESG1107), ES-Serum media: Knockout Dulbecco’s modified Eagle’s medium (DEMEM Gibco#10829-018), ES cell tested fetal calf serum (FCS), 2 mM glutamine, 1x Penicillin/Streptomycin, 1x non-essential amino acids, 110 nM ß-Mercaptoethanol, 1x nucleoside (100x Chemicon #ES-008D), 1000 U/ml LIF.

The cells were split with TrypLE Express (Thermo Fisher Scientific #12605-010) and the reaction was stopped with the same amount of phosphate-buffered saline (PBS Gibco #100100239) followed by centrifugation at 1000×*g* for 5 min. The cells were frozen in the appropriate media containing 10% dimethyl sulfoxide (DMSO, Sigma Aldrich #D5879). To minimize any effect of the 2i^34^ on the developmental potential mESC were only kept in 2i for the antibiotic selection for transgene integration after selection kept on feeders.

### Genetic manipulation of ES cells and generation of embryos and mice

ES cells were modified according to standard procedures. Briefly, 10 × 10^6^ ES cells were electroporated with 25 µg of linearized targeting construct and cultivated with selection media containing 250 µg/ml G418 (Life Technologies #10131035) or 125 µg/ml Hygromycin B (Life Technologies #10687010) for the first and second targeting, respectively. Resistant clones were isolated, and successful gene targeting was confirmed. Embryos and live animals were generated by tetraploid complementation^35^. Homozygous *Swthr*^*3xpA[N]/3xpA[H]*^ ES cells generated 21 mice from four foster mothers, confirming their integrity and usability in subsequent developmental assays. The selection cassettes consisting of *PGK::Neo-SV40pA* (abbreviated “N”) or *PGK::Hygro-SV40pA* (abbreviated “H”) were flanked by *FRT* sites. Selection cassette was removed by crossing animals with an FLP delete strain^36^.

### Generation of *Swthr* Rescue BAC

The BAC RP23-466K9 was ordered from BACPAC Resource Center (BPRC) and its integrity was verified by HindIII digest. The RP23-466K9 BAC contains the 175,271 bp of mouse genomic sequence surrounding the *Nkx2-5* locus. This BAC includes the upstream (*Bnip1*) and the downstream (*Kifc5b*) located genes. The BAC was modified using the Red/ET recombinase system (Genebridges). The H2B-Venus expression cassette, followed by a bglobin polyadenylation site and a downstream Neomycin selection cassette, was inserted into the ATG of *Nkx2-5*. This will eliminate any *Nkx2-5* expression from the BAC and simultaneously allows monitoring of rescue construct by means of detection of yellow fluorescence in the hearts of embryos and adults.

Around 3 Mio mESC cells of the C57Bl6J background were collected and resuspended in 680 µl PBS and were mixed with 120 µl linearized (PI-SceI) BAC (42 ng/µl). The BAC was electroporated into the C57Cl6J cells under the following conditions: 240 V; 500 µF; 4 mm; ∞ with a Gene Pulser Xcell™ Electroporation Systems from BioRad. Afterwards, the cells were resuspended in 2i Media and plated on gelatin-coated cell culture dishes. The next day the selection of the cells started with 300 µg/ml G418 (InvivoGen, #ant-gn-1). The selection was done till the colonies were big enough for picking after 7–8 days. Afterwards, the procedure was the same as described above.

### Generation of mouse embryos and strains from mESCs

All animal line generation procedures were conducted as approved by the Landesamt für Gesundheit und Soziales Berlin (LAGeSo), Berlin under the license numbers G0349/13. Embryos were generated by tetraploid morula aggregation of embryonic stem cells as described in ref. ^33^. SWISS mice were used for either wild-type donors (to generate tetraploid morula) or transgenic recipient hosts (as foster mothers for transgenic mutant embryos). All transgenic embryos and mESC lines were on a hybrid F1G4 (C57Bl6/129S6) background or the C57Bl6J background (rescue BAC). The parental cell line was a gift from A. Nagy^33^.

To generate the mouse strains the transgenic cells were aggregated with diploid morula SWISS embryos. The genotype of the cells was either hybrid F1 for the *Swthr* mutant mice or wild-type C57BL6J for the rescue mice. Adult *Swthr* mutant mice were backcrossed 6 times to C57BL6J before all subsequently conducted experiments. The *Swthr* strain was subsequently maintained by homozygous x homozygous breedings.

### Animal husbandry

Mice were housed at an artificial 12 h/12 h light-dark cycle. Fortified complete mouse feed (Ssniff #V1534-000) and water available *ad libitum*. The humidity and ambient temperature in the housing facility were controlled daily at a temperature of around 21 °C and a relative humidity of around 49%. All experimental procedures and maintenance were carried out in accordance with the Animal Welfare Act and the agreement of the responsible authorities (specifics given in the respective sections). Animals were monitored daily to ensure their well-being. Only male mice were used for AMI experiments and hence subsequent experiments. Breeding of animals was conducted for strain maintenance using homozygous  × homozygous breeding as they showed no phenotype and thus mouse numbers due to the absence of “wrong-genotype” littermates could be reduced. Only when animals were required for experiments, the breeding was increased to keep animal numbers low. Mice were euthanised by cervical dislocation for the collection of tissue.

### Whole-mount in situ hybridization

Whole-mount in situ hybridization was carried out using standard procedures described on the MAMEP website (http://mamep.molgen.mpg.de/index.php). Probes were generated by PCR from E11.5 heart ventricle cDNA using a primer containing promotor binding sites for T7 and SP6 polymerase. After verification of the probe templates, antisense in situ probes were generated as described on the MAMEP website using T7 polymerase (Promega #P2077). The in situ probes are generated against *Nkx2-5 or Sweetheart*.

### RNA isolation

To isolate RNA either from heart tissue or cultivated cardiomyocytes the cells were lysed in 900 µl Qiazol (Qiagen, #79306). To remove the DNA 100 µl gDNA Eliminator solution was added and 180 µl Chloroform (AppliChem, #A3633) to separate the phases. The extraction mixture was centrifuged at full speed, 4 °C for 15 min. The aqueous phase was mixed with the same amount of 70 % Ethanol and transferred to a micro or mini column depending on the amount of tissue and cells. The following steps were done according to the manufactural protocol.

### Subcellular RNA fractionation

Cellular fractionation was carried out as previously described^37^. Briefly, cell pellets were resuspended in 200 µl cold cytoplasmic lysis buffer (0.15% NP-40, 10 mM Tris pH 7.5, 150 mM NaCl) using wide orifice tips and incubated on ice for 5 min. The lysate was layered onto 500 µl cold sucrose buffer (10 mM Tris pH7.5, 150 mM NaCl, 24% sucrose w/v), and centrifuged in microfuge tubes at 16,000xg for 10 min at 4 C. The supernatant from this spin (700 µl) represented the cytoplasmic fraction. 10% (70 µl) of the supernatant volume was added to an equal volume of 2X sample buffer for immunoblot analysis. The remaining supernatant was quickly added to 15 ml tubes containing 3.5X volumes of QIAGEN RLT Buffer, supplemented with 0.143 M ß-mercaptoethanol. RNA purification from these and subsequent cellular fractions was performed according to manufacturer’s instructions.

The nuclear pellet was gently resuspended into 200 µl cold glycerol buffer (20 mM Tris pH 7.9, 75 mM NaCl, 0.5 mM EDTA, 50% glycerol, 0.85 mM DTT) using wide orifice tips. An additional 200 µl of cold nuclei lysis buffer (20 mM HEPES pH 7.6, 7.5 mM MgCl_2_, 0.2 mM EDTA, 0.3 M NaCl, 1 M urea, 1% NP-40, 1 mM DTT) was added to the samples, followed by a pulsed vortexing and incubation on ice for 1 min. Samples were then spun in microfuge tubes for 2 min at 19,000×*g* and at 4 °C. The supernatant from this spin represented the nucleoplasmic fraction (400 µl), and 10% of the supernatant was kept for immunoblot analysis. 3.5X volumes of QIAGEN RLT were added to the remaining nucleoplasmic supernatant.

50 µl of cold PBS was added to the remaining chromatin pellet, and gently pipetted up and down over the pellet, followed by a brief vortex. The chromatin pellet was extremely viscous and sticky, and therefore difficult to fully resuspend. 5 ml of the PBS supernatant was collected for immunoblot analysis as above, and 500 µl TRI-Reagent was added to the pellet. After vigorous vortexing to resuspend the chromatin, chromatin-associated RNA was extracted by adding 100 µl chloroform and incubated at room temperature for 5 min. The chromatin samples were then centrifuged in microfuge tubes for 15 min at 16,000×*g* at 4 C. The resulting upper aqueous layer was then added to 3.5X volumes of QIAGEN RLT buffer.

### Full-length cDNA determination

Rapid amplification of cDNA end (RACE) was performed using the SMARTer® RACE 5’/3’ Kit (Takara, #634858). 1 μg of freshly isolated RNA from E8.5 embryo hearts was used to generate first-strand cDNA according to the manufactural protocol. The primers were designed between 60–70 °C Tm. Half of the PCR product was analyzed by agarose gel electrophoresis and the rest was used for nested PCRs to validate the 5´and 3´end. PCR products were extracted from the agarose gel and sent for sequencing. After the determination of the end, the full-length sequences were amplified and sequenced. The sequences were deposited at GeneBank under the IDs: ON351017 (*Sweetheart RNA, Swhtr*).

### Fractionation of the main cell types of the adult heart

Fractionation was conducted as previously described^20^. Briefly, adult mice were sacrificed and hearts were collected in HBSS (gibco #14025050). Hearts were enzymatically digested using the Multi Tissue Dissociation Kit 2 (Miltenyi #130-110-203). Cardiomyocyte fraction was obtained by pre-plating. Endothelial cells and immune cells were obtained by magnetic separation. Fibroblasts were enriched by another preplating step.

### Cardiac injury models—ligation of the left anterior descending artery

Mouse experiments have been approved by the Government Veterinary Office (Service de la Consommation et des Affaires Vétérinaires—SCAV, Epalinges, Switzerland; License number: VD2027.3, VD3275) and were carried out in accordance with the institutional guidelines of the University of Lausanne, Lausanne, Switzerland, as well as Swiss laws concerning animal protection. For the procedure, the mouse was anesthetized by IP injection of a mixture of ketamin/xylazine/acepromazin (65/15/2 mg/kg). Mouse was placed on a warming pad for maintenance of body temperature. In the supine position, endotracheal intubation was performed, and the mouse was placed on artificial ventilation with a mini-rodent ventilator (tidal volume = 0.3 ml, rate = 120 breaths/min). Ocular gel was applied to hydrate the cornea during the surgical procedure. Proper intubation was confirmed by observation of chest expansion and retraction during ventilated breaths. A left thoracotomy was performed. The pectoralis muscle groups were separated transversely, and the fourth intercostal space was entered using scissors and blunt dissection. The pericardium was gently opened, and pressure was applied to the right thorax to displace the heart leftward. A 7.0 silk ligature was placed near the insertion of the left auricular appendage and tied around the left descending coronary artery. Occlusion of the artery was verified by the rapid blanching of the left ventricle. The lungs were re-expanded using positive pressure at end-expiration and the chest and skin incision were closed respectively with 6-0 and 5-0 silk sutures. The mouse was gradually weaned from the respirator. Once spontaneous respiration resumed, the endotracheal tube was removed, and the animal was replaced in his cage on a warming pad with standard chow and water ad libitum. Analgesic drug (Temgesic, Buprenorphin 0.1 mg/kg) was administered subcutaneously after the surgery.

Animal experiments were approved by the Government Veterinary Office (Lausanne, Switzerland) and performed according to the University of Lausanne Medical School institutional guidelines.

### In vivo transthoracic ultrasound imaging

Mouse experiments have been approved by the Government Veterinary Office (Service de la Consommation et des Affaires Vétérinaires—SCAV, Epalinges, Switzerland; License number: VD2027.3, VD3275) and were carried out in accordance with the institutional guidelines of the University of Lausanne, Lausanne, Switzerland, as well as Swiss laws concerning animal protection. Transthoracic echocardiography was performed using a 30 MHz probe and the Vevo 2100 Ultrasound machine (VisualSonics, Toronto, ON, Canada). Mice were lightly anesthetized with 1–1.5% isoflurane, maintaining heart rate at 400–500 beats per minute. The mice were placed in decubitus dorsal on a heated 37 °C platform to maintain body temperature. A topical depilatory agent was used to remove the hair and ultrasound gel was used as a coupling medium between the transducer and the skin. The heart was imaged in the 2D mode in the parasternal long-axis view. From this view, an M-mode cursor was positioned perpendicular to the interventricular septum and the posterior wall of the left ventricle at the level of the papillary muscles. Diastolic and systolic interventricular septum (IVS;d and IVS;s), diastolic and systolic left ventricular posterior wall thickness (LVPW;d and LVPW;s), and left ventricular internal end-diastolic and end-systolic chamber (LVID;d and LVID;s) dimensions were measured. The measurements were taken in three separate M-mode images and averaged. Left ventricular fractional shortening (%FS) and ejection fraction (%EF) were also calculated. Fractional shortening was assessed from M-mode based on the percentage changes of left ventricular end-diastolic and end-systolic diameters. %EF is derived from the formula of (LV vol;d – LV vol;s)/ LV vol;d*100. Echographies were done in baseline condition and one and two weeks after surgery. Sacrifices were done on the day of the 2-week post-MI echography.

### Heart preparation and histology

Adult hearts were dissected two weeks after MI and fixed in 4% paraformaldehyde/PBS overnight. Fixed hearts were embedded in paraffin and sections (4–6 µm thickness) were mounted onto Superfrost® Plus microscope slides (Thermo Scientific #630-0950). Immunohistochemistry was carried out using standard procedures. The Antibody used for the detection of cell borders was anti-wheat germ agglutinin (WGA, Thermo Fisher Scientific, #W11261). The slides were mounted with Vectashield (VWR, #101098-042) and sealed with colorless nail polish. Image documentation was conducted using the NIKON Eclipse Ci, equipped with the Ds-Ri2 color camera. Analysis of cell sizes was conducted using ImageJ software.

### Real-time quantitative PCR analysis

Quantitative PCR (qPCR) analysis was carried out on a StepOnePlus^TM^ Real-Time PCR System (Life Technologies) using Power SYBR® Green PCR Master Mix (Promega #A6002). RNA levels were normalized to housekeeping genes. Quantification was calculated using the ΔΔCt method^38^. *Hmbs* served as a housekeeping control gene for qPCR. The primer concentration for a single reaction was 250 nM. Error bars indicate the standard error from biological replicates, each consisting of technical duplicates. A list of oligonucleotides can be found in Table S1.

### Embryo/heart preparation and histology

Staged embryos and adult hearts were dissected from uteri into PBS and fixed in fresh 4% paraformaldehyde/PBS 1 mm tissue per 1 h at 4 °C. For histology, embryos and hearts were embedded in paraffin. E7.5 embryos were removed from the uterus of timed mated mothers together with the surrounding decidua and fixed together. Sections (4–6 µm thickness) were mounted onto Superfrost® Plus microscope slides (Thermo Scientific) or on Zeiss MembraneSlide 1.0 PEN NF (#415190-9081-001). The stainings were carried out with Eosin (Carl Roth), Hematoxylin (AppliChem), and Sirius Red according to standard procedures. All image documentation was carried out on a Microscope Leica M205C with the MC170 HD camera and captured with ImageJ. Except for the E7.5 embryo sections, which were imaged on a NIKON Eclipse Ci, equipped with the Ds-Ri2 color camera.

### SmFISH

FISH Probes were designed, using the biosearchtech.com/stellaris-designer website and ordered from BioCat. The lyophilized Probes were resuspended in 400 µl 1x Tris–EDTA Buffer (10 mM) Tris–HCl (ApliChem, #A1086, ApliChem, #A5634), 1 mM EDTA (Life Technologies, #15575020), pH 8.0 to get a final concentration of 500 nM per µl. The probes were conjugated with a Quasar570 dye and small aliquots were stored at −20 °C.

Cardiomyocytes from P0 were seeded on coverslips (10 mm Marienfeld, #0111500) and left to attach overnight. Subsequently, they were cultured under specified conditions for 24 h. For the fixation process, the cells were washed twice with PBS and fixed for 10 min at room temperature with 3.7% PFA/PBS (AppliChem, #A3813). Again, the cells were washed 3 times with PBS and immersed in 70% EtOH for 1 h at 4 °C. Prior to hybridization the coverslips were washed with diluted wash buffer A (LGC Biosearch Technologies #SMF-WA1–60) containing 10% deionized formamide (Merck #F9037). The coverslips were immersed in a hybridization buffer (LGC Biosearch Technologies #SMF-HB1-10) containing 10% deionized formamide and 1:100 smRNA FISH probe (Stellaris) and incubated at 37 °C o/n. After washing the coverslips at 37 °C for 30 min in the dark, they were washed again adding Hoechst DNA stain (Thermo Fisher Scientific #H3570) at 37 °C for 30 min in the dark. The coverslips were equilibrated in glucose buffer (2x SSC, 0.4% glucose, 20 mM Tris–HCl pH 8.0) and subsequently incubated in glucose buffer supplemented with 1:100 glucose oxidase and catalase. For mounting ProLong™ Gold Antifade Mountant (Life Technologies #P36930) was used. Images were acquired using a Nikon ECLIPSE Ti2 widefield microscope with a Nikon Plan Apo λ ×100/1.45-numerical aperture oil objective lens and a Nikon DS-Qi2 camera. Z-stacks of 200 nm step size were acquired and used to create maximum intensity projections by ImageJ/Fiji software. FISH signals were quantified manually using ImageJ/Fiji.

### Heart slice preparation, treatment and harvest

4 Wild-type and 4 *Swhtr*^*3xpA/3xpA*^ mice were sacrificed by cervical dislocation when they reached the age of 8 weeks. The heart was collected in HBSS and washed in BDM buffer (HBSS + 10 mM BDM) to remove blood cells. Apex and base of the heart were manually removed before preparing the slices using a sharp scalpel. The slices were washed in BDM buffer once more before submerging them in a culture medium consisting of DMEM (gibco#10569010) supplemented with 10% fetal bovine serum, 1% non-essential amino acids (gibco #11140050) and 1% Penicillin–Streptomycin (gibco #15140122). The heart slices were left to recover overnight. During the experiment, the media was refreshed every other day.

For the treatment, the slices were incubated in a humidified hypoxic chamber (3% O_2_, 5% CO_2_) at 37 °C for 7 days and moved to a humidified incubator with normoxic conditions (21% O_2_, 5% CO_2_) at 37 °C for 7 additional days. Slices that were incubated in a humidified incubator at normoxic conditions (21% O_2_, 5% CO_2_) at 37 °C for 14 days served as the control. Following the treatment, the slices were harvested in 1 ml TRI reagent (Sigma-Aldrich #T9424) and homogenized using Precellys® 2 ml Soft Tissue Homogenizing Ceramic Beads. RNA was extracted by Phenol/Chloroform extraction. Briefly, 200 µl chloroform was added per 1 ml of TRI reagent and mixed. After centrifugation at 4 °C aqueous phase was precipitated by adding 0.7 vol of Isopropanol and incubating at –20 °C for 1 h. The precipitated RNA was pelleted by centrifugation at 4 °C, washed with 70% ethanol, and resuspended in nuclease-free water.

### Growth and care of HL-1 cardiomyocytes

The HL-1 cardiomyocytes (Sigma-Aldrich # SCC065) were a kind gift from Prof. Dr. Ralf Gilsbach (Institute of Experimental Cardiology, Heidelberg University Hospital). HL-1 cardiomyocytes were maintained in a humidified CO_2_ incubator at 37 °C and 5% CO_2_ and cultured in Claycomb medium (Thermo Fisher Scientific #51800C) supplemented with 10% fetal bovine serum (PAN-Biotech #P30-2600), 1% Penicillin–Streptomycin (gibco #15140122), 1% GlutaMAX™ Supplement (gibco #35050061), and 0.1 mM Norephinephrine (Sigma-Aldrich #A7257). Culture dishes were coated with 0.1% Gelatin (Sigma-Aldrich #G1393) containing 5 µg/ml Fibronectin (Merck #341631) for 30 min at 37 °C in advance to introducing cells.

For passaging, the culture medium was removed completely and the cells were washed with DPBS (gibco #14190144) once to remove the serum. The cells were incubated with Trypsin–EDTA (gibco #25300054) for 5–10 min at 37 °C. The cells were collected in DPBS and centrifuged at 1000×*g* for 5 min. The cell pellet was resuspended in a Claycomb full medium and seeded in the required density.

### Transfection of HL-1 cardiomyocytes

For transfection HL-1 cardiomyocytes were detached and cell culture plates coated as described above. For LNA GapmeR (QIAGEN) mediated knockdown a reagent-aided approach using Lipofectamine™ RNAiMAX transfection reagent (Thermo Fisher Scientific #13778150) was conducted following the manufacturer’s guidelines with experimentally determined optimizations. For 3*10^5^ cells 5 µl of Lipofectamine™ RNAiMAX transfection reagent were diluted in Opti-MEM (gibco #11520386). Separately, the LNA GapmeRs (QIAGEN #339511) were diluted in Opti-MEM to achieve a final concentration of 20 nM. The diluted reagents were mixed and incubated at room temperature for 15 min. After the incubation, the coating solution was aspirated from the prepared cell culture dishes, and the transfection mix was added. Detached HL-1 cells were resuspended in Opti-MEM containing 5% fetal bovine serum (PAN-Biotech #P30-2600) and 1% Penicillin–Streptomycin (gibco #15140122) and counted using the Countess™ 3 Automated Cell Counter. The cells were seeded on top of the transfection media in a density of 1.5*10^5^ cells/ml and left to attach. After 4 h the medium was changed to Claycomb full medium.

### RNA immunoprecipitation

Magnetic beads (Thermo Fisher Scientific #88803) were washed in binding buffer (10 mM Tris–HCl, 150 mM NaCl, 1% NP-40, 0.5% Triton-X, 1 mM EDTA) three times and coupled to 5 µg of the NKX2-5 antibody (R&D Systems #AF2444 Lot UQW0120031) for 1 h at room temperature on a rotating wheel. The same procedure was executed for the normal goat IgG negative control (R&D Systems #AB-108-C Lot ES4521111). Subsequently, the beads were washed with binding buffer three times and kept on ice until further use.

HL-1 cardiomyocytes were seeded on 10 cm to 80% confluency and subsequently incubated under normoxic or hypoxic conditions for 24 h. Medium was removed and cells were washed with DPBS (gibco #14190144) before crosslinking (Stratagene UV Stratalinker 1800) at 100 mJ. Two 10 cm dishes per condition were scraped into ice-cold DPBS, washed and resuspended in lysis buffer (10 mM Tris–HCl, 150 mM NaCl, 1% NP-40, 0.5% Triton-X, 1 mM EDTA, 5% Glycerol) supplemented with 1x Protease Inhibitors (Roche #11873580001). After 20 min incubation on ice, the lysate was homogenized using a Kimble Dounce Glass homogenizer and cleared by centrifugation. The clear lysate was added to pre-coupled beads and incubated on a rotating wheel at 4 °C o/n. The beads were washed 2 times with binding buffer, one time with high salt buffer (20 mM Tris–HCl pH 7.5, 500 mM NaCl, 10 mM EDTA) and LiCl buffer (10 mM Tris–HCl pH 8.0, 250 mM LiCl, 0.5% NP-40, 1% sodiumdeoxycholate) followed by two washes with binding buffer. For qRT-PCR analysis the beads were resuspended in Protein degradation buffer (200 mM Tris–HCl pH 8.0, 300 mM NaCl, 25 mM EDTA, 2% SDS, 6.4 U Proteinase K (NEB #P8107S)) and incubated at 50 °C for 30 min at 450 rpm. After the addition of TRI Reagent (Thermo Fisher Scientific #93289) the samples were used for phenol–chloroform extraction and analyzed as described above. For Western Blot verification of successful protein enrichment, the beads were resuspended in 1x reducing NuPAGE™ LDS Sample Buffer (Invitrogen #NP0007 + #NP0004) and analyzed by SDS–PAGE (Invitrogen #NP0321BOX, #NP0001, #NP0006) using PVDF membrane (Merck #3010040001). For visualization the proteins were detected with a different antibody for pulldown to reduce IgG background (Cell Signaling #5444 Lot 4 (1:1000), #7074 (1:500)) and visualized using Pierce™ ECL Western Blotting-Substrate (Thermo Fisher Scientific # 32109) in an Azure 300 Darkroom Eliminator (Azure Biosystems # 513001).

### Differentiation of human iPSCs

Commercially available human iPSCs (WTSIi081-A, EBiSC #66540196) were maintained in a humidified CO_2_ incubator at 37 °C and 5% CO_2_ and cultured in mTeSR™ Plus supplemented media (STEMCELL #100-0276) on Vitronectin XF™ (STEMCELL #07180) coated plates (0.5 µg/cm^2^). Cells were routinely passaged using Accutase® (Sigma-Aldrich #A6964) for detachment and subsequently seeded in an appropriate density. For differentiation into cardiomyocytes, the STEMdiff™ Ventricular Cardiomyocyte Differentiation Kit (STEMCELL #05010) was used following the manufacturer’s guidelines. The ethics board of the Goethe University, Faculty of Medicine, approved the use of the iPSC line for this study under the approval number 2023-1503-Anfrage.

### Bioinformatic analysis and data deposit

RNA was treated to deplete rRNA using Ribo-Minus technology. Libraries were prepared from purified RNA using ScriptSeq™ v2 and were sequenced on an Illumina novaseq 6000 platform at Novogene. We obtained 25 million paired-end reads of 150 bp length. Read mapping was done with STAR aligner using default settings with the option --outSAMtype BAM SortedByCoordinate^39^ with default settings. For known transcript models we used GRCm38.102 Ensembl annotations downloaded from Ensembl repository^40^. Counting reads over gene model was carried out using GenomicAlignments Bioconductor package^41^. The data are deposited to GEO under the accession number GSE200380.

All genes with read counts <10 were excluded. For normalization of read counts and identification of differentially expressed genes, we used DESeq2 with Padj <0.05 and log2FC = 0.58 cutoff^42^. GO terms were analyzed using clusterProfiler and enrichplot Bioconductor packages^43^. To overlap NKX2-5 binding with *Swhtr*-dependent DE genes, we downloaded ChIP-sequencing data from adult heart apex tissue (GSM3518668). Raw reads were downloaded and aligned to mm10 using Bowtie2^44^. Samtools^45^ was used to convert aligned reads to sorted bam files. Duplicated reads as well as reads overlapping blacklisted regions were removed using bedtools^46^. Peaks were called with MACS3 peak caller^47^. All peaks were sorted and merged using bedtools. DE gene’s genomic coordinates were extracted using GenomicFeatures package then gene coordinates were extended to 1000 bp on both sides. Finally, peaks are intersected with gene coordinates using the IRanges Bioconductor package^48^. The *p*-value of the overlapping Granges was calculated according to the hypergeometric test implemented in the gmp R-CRAN package.

### Reporting summary

Further information on research design is available in the Nature Portfolio Reporting Summary linked to this article.

### Supplementary information

Supplementary Information

Peer Review file

Reporting Summary

### Source data

Source Data

## Data Availability

The RNA-sequencing data of WT and *Swhtr Null* cardiac slices following hypoxia and recovery are deposited to GEO and can be downloaded under the accession number GSE200380. The NKX2-5 ChIP-sequencing data from adult heart apex tissue^21^ used is publicly available at GEO under the accession number GSM3518668. The RNA-sequencing tracks from hiPSC-derived cardiomyocytes are publicly available at ENCODE^32^ under the accession number ENCSR379YAE. The cDNA of *Sweetheart RNA* (*Swhtr*) is deposited with GenBank under ON351017. Source data are provided with this paper.
