# Supplementary material for: The lncRNA Sweetheart regulates compensatory cardiac hypertrophy after myocardial injury in murine males
Source: Nat Commun. 2023 Nov 2;14:7024. doi: 10.1038/s41467-023-42760-y (PMC10622434; doi:10.1038/s41467-023-42760-y)
Supplement: Supplementary file 1 — Supplementary Information [file 41467_2023_42760_MOESM1_ESM.pdf]

Supplementary Information

The lncRNA *Sweetheart* regulates compensatory cardiac hypertrophy after myocardial injury in murine males

Sandra Rogala[1,2], Tamer Ali[1,2,3], Maria-Theodora Melissari[1], Sandra Währisch[4], Peggy Schuster[1], Alexandre Sarre[5], Rebeca Cordellini Emídio[1], Thomas Boettger[6], Eva-Maria Rogg[1], Jaskiran Kaur[1], Jaya Krishnan[1], Gabrijela Dumbović[1], Stefanie Dimmeler[1], Samir Ounzain[7,8], Thierry Pedrazzini[7], Bernhard G Herrmann[4] and Phillip Grote[1,2,9*]

[1] Institute of Cardiovascular Regeneration, Centre for Molecular Medicine, Goethe University, Theodor-Stern-Kai 7, 60590 Frankfurt am Main, Germany
[2] Georg-Speyer-Haus, Institute for Tumor Biology and Experimental Therapy, Paul-Ehrlich-Str. 42-44, 60596 Frankfurt am Main, Germany
[3] Faculty of Science, Benha University, Benha 13518, Egypt
[4] Department of Developmental Genetics, Max Planck Institute for Molecular Genetics, Ihnestr. 63–73, 14195 Berlin, Germany
[5] Cardiovascular Assessment Facility, University of Lausanne Medical School, Lausanne, Switzerland
[6] Department of Cardiac Development and Remodelling, Max Planck Institute for Heart- and Lung Research, 61231 Bad Nauheim, Germany
[7] Experimental Cardiology Unit, Department of Cardiovascular Medicine, University of Lausanne Medical School, Lausanne, Switzerland
[8] HAYA Therapeutics, Rte de la Corniche 6, 1066 Lausanne, Switzerland
[9] Frankfurt Cancer Institute, Goethe University Frankfurt, Frankfurt am Main, Germany

Corresponding author name(s): Dr. Phillip Grote, ph.grote@Georg-speyer-haus.de

<u>List of Supplementary Material:</u>

Supplementary figures S1 to S6

Supplementary table S1

Rogala, Supplementary Figure 1

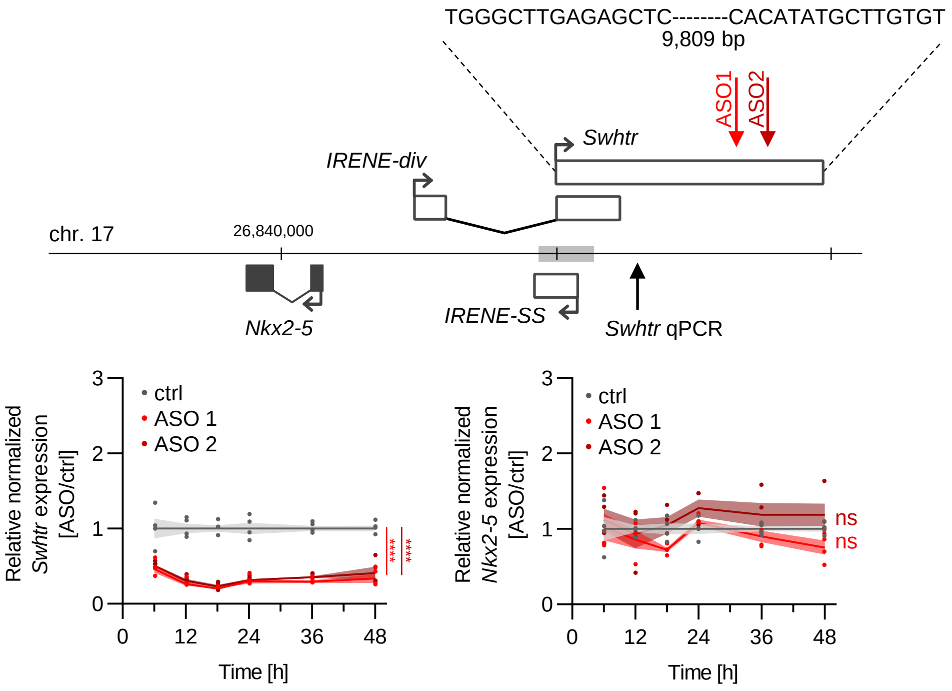

Supplementary Figure 1. **Presence of full length *Swhtr* in HL-1 cardiomyocytes.** Outline of the genomic locus. Depicted are the *Swhtr* 5' and 3' ends obtained by RACE-PCR. GapmeR mediated knockdown of *Swhtr* using two different LNA GapmeRs (ASO 1 and ASO 2) targeting the *Swhtr*-3' region, measured with qRT-PCR using 5' located primers as indicated above. Statistical significance assessed by Two-way ANOVA using multiple comparisons with Tukey correction for each timepoint and indicated by asterisks in the respective color. ns = not significant, **** < 0.0001 (n=4)

Rogala, Supplementary Figure 2

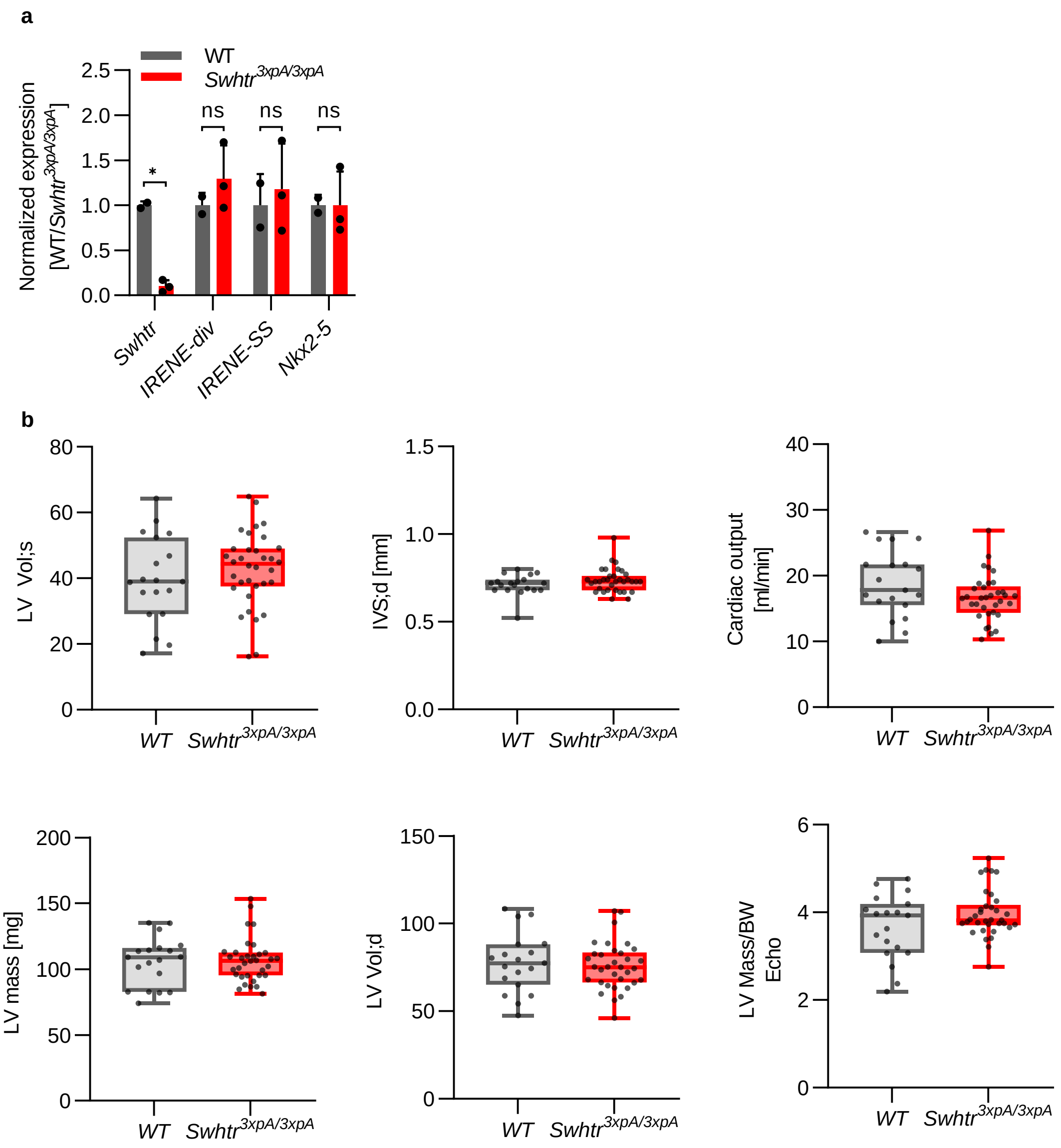

Supplementary Figure 2. **No difference in heart parameters in *Swhtr*$^{3xpA/3xpA}$ mutant mice. a** qRT-PCR analysis of the *IRENE* RNAs in *Swhtr*$^{3xpA/3xpA}$ mouse hearts. Number of dots indicates number of replicates. **b** Additional heart parameters assessed by echocardiography. No statistically significant differences were detected.Statistical significance was tested by Two-way ANOVA with Tukey correction in case of multiple comparisons. ns = not significant, * < 0.05

Rogala, Supplementary Figure 3

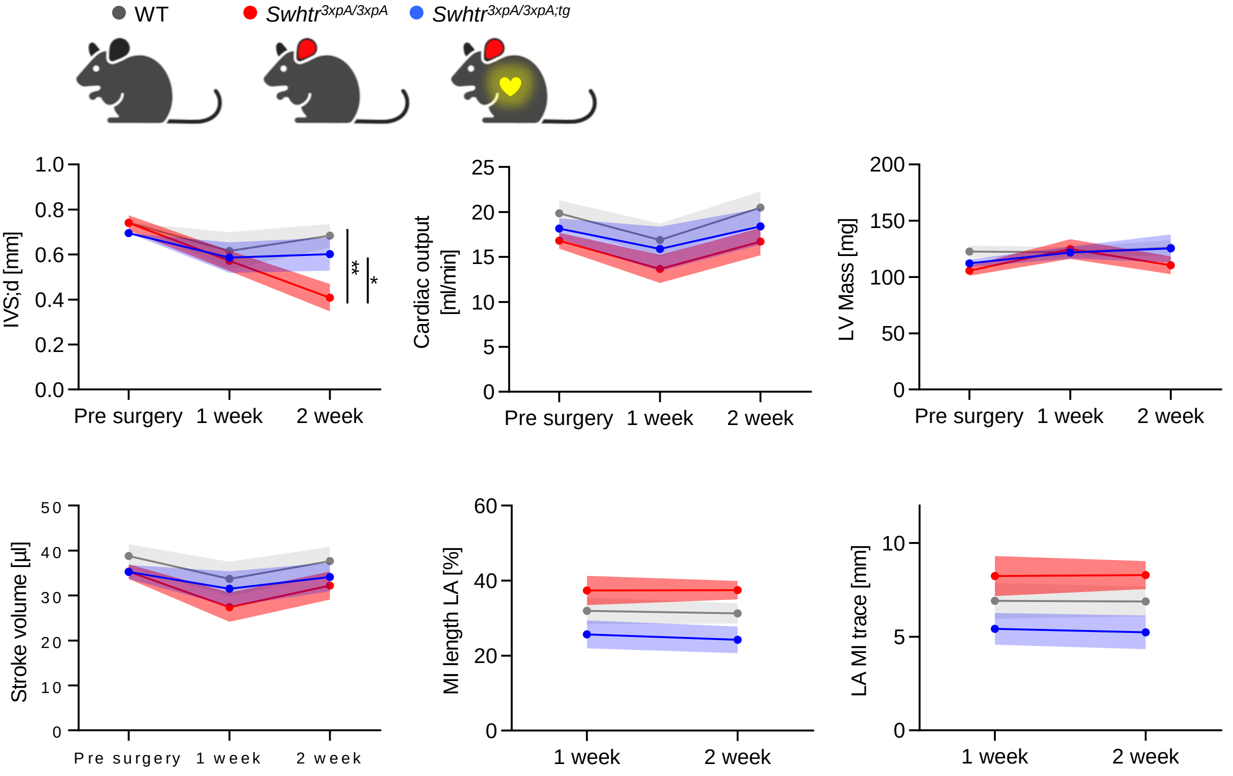

Supplementary Figure 3. **Additional heart parameters after MI.** Statistical significance tested by Two-way ANOVA with Tukey correction for multiple comparisons. No indication implies that no statistically significant difference from the wild type was detected. * < 0.05, ** < 0.01 (n=9 animals per genotype). The color key for the genotype was generated using BioRender.com.

Rogala, Supplementary Figure 4

a

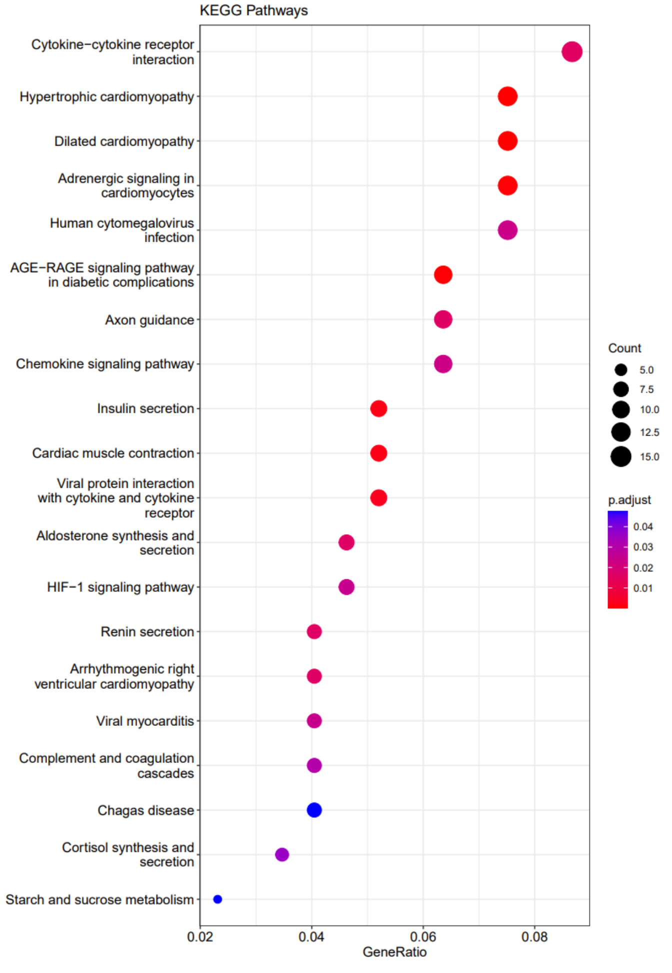

b

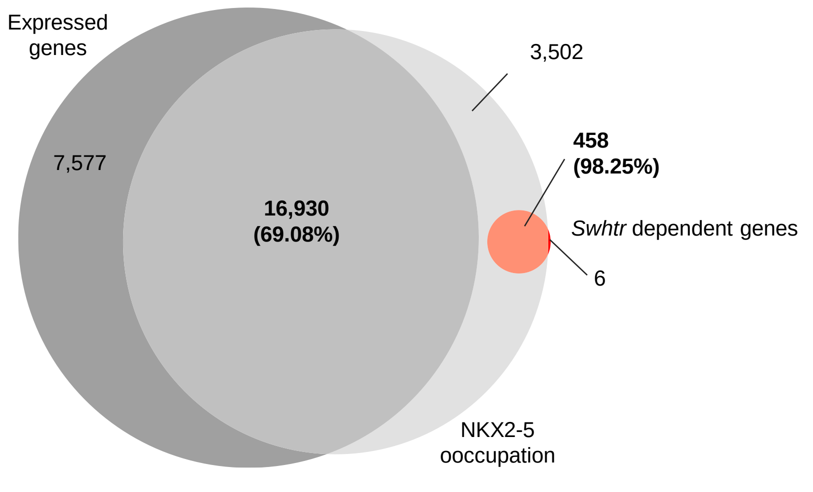

Supplementary Figure 4. **KEGG-pathway-analysis and NKX2-5 occupation. a** KEGG pathway analysis of of *Swhtr* dependent genes (464) **b** VENN diagram of all expressed genes without dysregulated genes (dark grey), NKX2-5 occupied gene bodies (light grey) and the 464 *Swhtr* dependent genes (red). The p-value for the *Swhtr* dependent genes is 2.780948e-32 by hyper geometric test gmp.

Rogala, Supplementary Figure 5

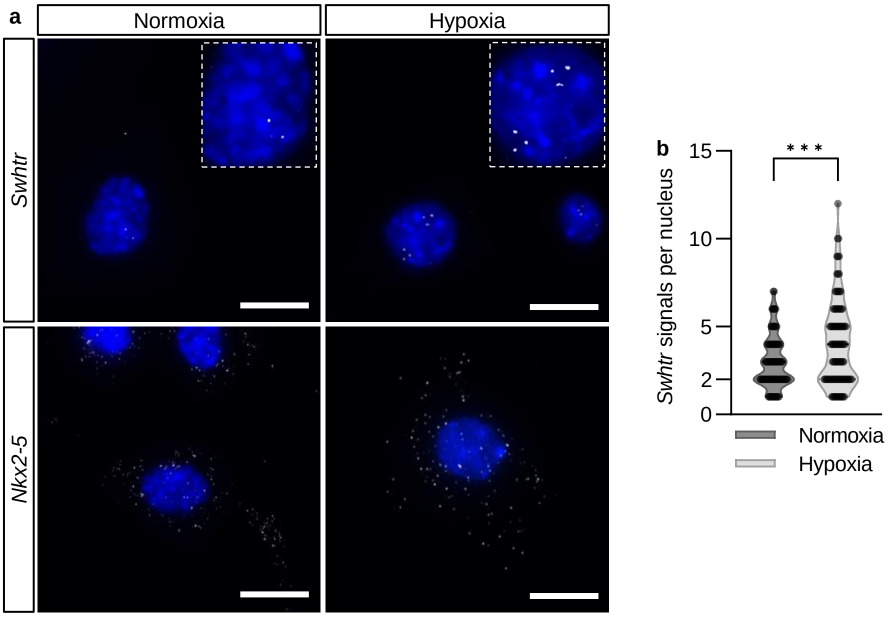

Supplementary Figure 5. ***Swhtr* dispersion under hypoxic stress in HL-1 cardiomyocytes. a** smFISH stainings of *Swhtr* and *Nkx2-5* in HL-1 cardiomyocytes after 24h incubation at normoxic (21% O_2) or hypoxic (1% O_2) conditions. The white line represents 10 µm. **b** Quantification of *Swhtr* smFISH signals in the nucleus of HL-1 cardiomyocytes after 24h incubation at normoxic (21% O_2) or hypoxic (1% O_2) conditions (n > 50). Statistical significance tested by Two-way ANOVA. *** < 0.001

Rogala, Supplementary Figure 6

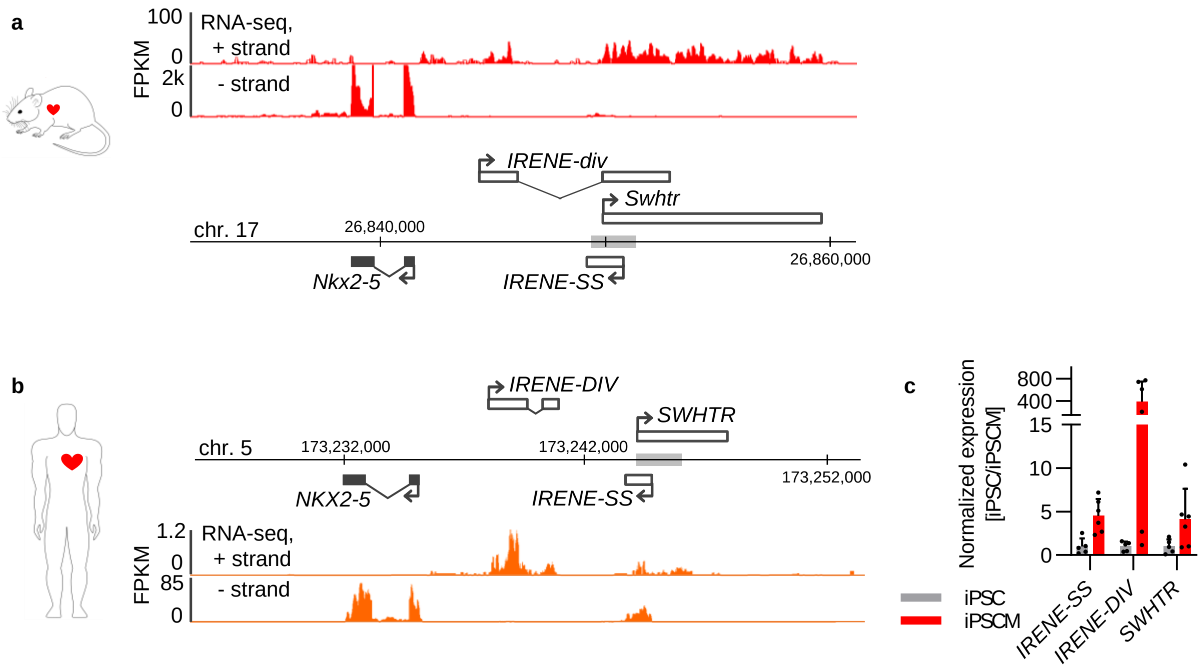

Supplementary Figure 6. **Conservation between human and mouse. a** Murine strand-specific RNA sequencing data from the heart generated by us depicting the annotation of the indicated RNAs. **b** Human strand-specific RNA sequencing data from cardiomyocytes from public ENCODE datasets (ENCFF182KCL, ENCFF571PYD) depicting RNA-sequencing derived annotations of the indicated RNAs. **c** qRT-PCR validation of presence of the *NKX2-5* associated lncRNAs in cardiomyocytes (iPSCM) derived from induced pluripotent stem cells (iPSCs) as compared to the undifferentiated condition (iPSC n = 5; iPSCM n = 6).

Table S1 – related to Materials & Methods

Oligos and probes used for indicated experiments

qPCR analysis	Forward	Reverse
Nkx2-5	AAGTGCTCTCCTGCTTTCCCA	TTTGTCCAGCTCCACTGCCTT
Swhtr	CCGCAAACCGAACAACTCAG	CTGCCTAAGTGGGGGAAGTG
Eaf1	agagctgtggtgttggcatt	agagagcaaaacctggagcc
Tnni1	CCATGGATCTGCGGGCCAACC	GCGGCCTTCCATGCCAGACA
Cdh5	TTGGACCGAGAGAAACAGGC	CTTGCCCACTCGGATGTCTT
Ptprc	GGCTTCAAGGAACCCAGGAA	GAGTGCCTTCCTCCATGCTT
Ddr2	AGCGAGGTACAGGACTCCAT	TCCCCGCTCCTCCTCAATTA
Xist	agggtgtgtgtgcatatgga	ccgccatcttttcctgtacg
Snrnp70	tgctcctcctccaacaagag	cagagtcttgaaggcatcgc
Hmbs	CCTGGGCGGAGTCATGTC	ACTCGAATCACCCTCATCTTTGA
IRENE-div	TGGGAAAGGAGTGTGTGTCTG	GGAGGACCTTGTTACTGGCA
IRENE-SS	TCTCTTTGGTCACTTGGGGC	GCCGCCGTTTATGCTGTAG
hSWHTR	GAGAGGGCAGCTTGGACTTT	CTTCTTTCCCCTCTGCCCTG
hIRENE-DIV	TCGCTTTCAGACTTCCGGTG	CCAGCGTCTCGATTCTCTCC
hIRENE-SS	TCAGCTGGCACCAAATCCTT	CAGTCTCAGCTGTGCAGTCA

RACE analysis	Reverse
5´ *Swhtr*	GATTACGCCAAGCTTTCTCCATCCCGCTTTTCACC
3´ *Swhtr*	GATTACGCCAAGCTTATCCCCTGGAACTGGGGTTA

smFISH probes	*Nkx2-5*	*Swhtr*
1	ctggagtagggggggattcag	accacttacaacctgattca
2	caggtgggtagcagagagtg	atgtgcaccttgaaagcttg
3	gagaaaggcgtgggtgtgag	aaatgctccttttaagggct
4	caggttcaggatgtctttga	gttcacactaattggtgtgc
5	gaaagcaggagagcacttgg	aaaggccctatcgattactt
6	taggctcccgggtaaaatgt	gaagtggagcagttgaagca
7	gcgcacagctctttttttatc	caaagagtatctgggcctac
8	ctgcgagaagagcacgcgtg	gtattgcagccaagaagtga
9	ggtaccgctgttgcttgaag	ggcaattggaagggagcaag
10	tggacgtgagcttcagcacg	ctcagcagtttgagtaatcc
11	tggaaccagatcttgacctg	ctactttgtttccgtacaga

12	tcgcttgcacttgtagcgac	aacctttttgctattgccaag	
13	agaagctccagagtctggtc	cctgaagggaaggactagtt	
14	ttgtagccataggcattgag	ctgaacactctgcctaagtg	
15	gacgccaaagttcacgaagt	tgttagagggaaagacaccc	
16	gactctgcacggtgttcaag	taggacaggaagagagccat	
17	ttctcctaaaggtgggagtc	gaaaaggtctaagtgtccct	
18	tgggtgtgaaatctgaggga	ttaccaaccaggcactatag	
19	gggatggatcggagaaaggt	gtcacagagatagagcagga	
20	acaccaggctacgtcaataa	gtgttctttcgttcagtttg	
21	gtgtggaatccgtcgaaagt	ctagttttcgggtcaagagt	
22	cgacggcaagacaaccaggg	tcatgaacccacattgcata	
23	aacataaatacgggtgggtg	gtcaagaatgctcaacctgg	
24	gagggttctcatttcttaca	gtttcagactgtaggcattt	
25	gttagcgcactcactttaat	ctttccatttgataggcaga	
26		gggatacttgcctacatatt	
27		tatccctttggattttgagt	
28		ccatccagaatggatagagt	
29		aaatccatagcattggctgg	
30		atagctcgattctctaggtg	
31		caacctcaaggccagaagaa	
32		agactttccttggttgaagg	
33		aaatcgcttagactgcgtgg	
34		gacccaaaacaacagccatg	
35		ctagttcttaccaccaagta	
36		gcccaatagttagaacatgc	
37		ctagaaaccaaagccctact	
38		ttgtcctgtggacttaacac	
39		tcctggtgctcaaggtaaaa	
40		gatgtccaggatggaaatgc	
41		aatacattggaacaccctgc	
42		ttccaatctgtgcagaagtc	
43		aggcccaggtaaaagaactc	
44		ttgttatctattgtgccacg	
45		atgcattactatatagccca	
46		attcacatttatcctgacct	
47		tatcttattccatgctcact	
48		tgccaactgaaccagtttta	
Genotyping	Forward	Reverse	Internal
Swhtr3xpA/3xpA	CCCGCATGAAGATTCTGGGA	GCCTGAAATGAGCCTTGGGA	CAGGTTGGAAGTGGAGCAG

Swhtr3xpA/3xpA;tg	TGACCGAGTACAAGCCCAC	GCGCAGACAGGTCCCCAGAC

WISH probes	Forward	Reverse
Nkx2-5	aATTTAGGTGACACTATAGAA	aTAATACGACTCACTATAGG
	CAAGTGCTCTCCTGCTTTCC	GTGGAATCCGTCGAAAGT
Swthr	aATTTAGGTGACACTATAGAA	aTAATACGACTCACTATAGG
	AATCTTTGGGCCAGGACTTT	TCCACCTCTTTTTCCATTGC

Antisense LNA GapmeRs	Sequence
Swhtr ASO1	TAGGATTGAGAGACGT
Swhtr ASO2	TTCGATGGATCATTAA
GapmeR negative control A	AACACGTCTATACGC
