# Supplementary material for: The lncRNA Sweetheart regulates compensatory cardiac hypertrophy after myocardial injury in murine males
Source: Nat Commun. 2023 Nov 2;14:7024. doi: 10.1038/s41467-023-42760-y (PMC10622434; doi:10.1038/s41467-023-42760-y)
Supplement: Supplementary file 3 — Reporting Summary [file 41467_2023_42760_MOESM3_ESM.pdf]

nature portfolio

Corresponding author(s): Phillip Grote

Last updated by author(s): Oct 13, 2023

Reporting Summary

Nature Portfolio wishes to improve the reproducibility of the work that we publish. This form provides structure for consistency and transparency in reporting. For further information on Nature Portfolio policies, see our Editorial Policies and the Editorial Policy Checklist.

Statistics

For all statistical analyses, confirm that the following items are present in the figure legend, table legend, main text, or Methods section.

n/a	Confirmed	
☐	☒	The exact sample size (*n*) for each experimental group/condition, given as a discrete number and unit of measurement
☐	☒	A statement on whether measurements were taken from distinct samples or whether the same sample was measured repeatedly
☐	☒	The statistical test(s) used AND whether they are one- or two-sided<br>*Only common tests should be described solely by name; describe more complex techniques in the Methods section.*
☐	☒	A description of all covariates tested
☐	☒	A description of any assumptions or corrections, such as tests of normality and adjustment for multiple comparisons
☐	☒	A full description of the statistical parameters including central tendency (e.g. means) or other basic estimates (e.g. regression coefficient) AND variation (e.g. standard deviation) or associated estimates of uncertainty (e.g. confidence intervals)
☐	☒	For null hypothesis testing, the test statistic (e.g. *F*, *t*, *r*) with confidence intervals, effect sizes, degrees of freedom and *P* value noted<br>*Give P values as exact values whenever suitable.*
☒	☐	For Bayesian analysis, information on the choice of priors and Markov chain Monte Carlo settings
☒	☐	For hierarchical and complex designs, identification of the appropriate level for tests and full reporting of outcomes
☒	☐	Estimates of effect sizes (e.g. Cohen's *d*, Pearson's *r*), indicating how they were calculated

Our web collection on statistics for biologists contains articles on many of the points above.

Software and code

Policy information about availability of computer code

Data collection	Public data that were used in this study were download from GEO. The access number for each dataset is given. Quantitative PCR data were collected using the provided Software StepOne. Fluorescent image data on the Nikon Ti2 was obtained with the provided NIS-Elements software and analyzed using ImageJ/Fiji. Statistics were analyzed using the GraphPad 9 software. Ultrasound on mice were taken on a Vevo 2100 Ultrasound machine and data analzed with the provided VevoStrain software.
Data analysis	All code used in this study is publicly available.<br>RNA-seq data were aligned using STAR aligner using default settings with the option --outSAMtype BAM SortedByCoordinate. GRCm38.102 Ensembl annotations were used as transcript models. The GenomicAlignments Bioconductor package was used to calculate the read count. Differential genes were called using DESeq2 with Padj < 0.05 and log2FC= 0.58 cutoff. GO terms were analyzed using clusterProfiler and enrichplot Bioconductor packages.<br>For the overlapp calculation with dysregulated genes the downloaded data (GSM3518668) were aligned to mm10 using Bowtie2. Samtools was used to convert aligned reads to sorted bam files. Duplicated reads as well as reads overlapping blacklisted region were removed using bedtools. Peaks, were called with MACS3 peak caller. All peaks were sorted and merged using bedtools. DE genes genomic coordinates were extracted using GenomicFeatures package then genes coordinates were extended 1000 bp on both sides. Finally, peaks are intersected with genes coordinates using the IRanges Bioconductor package.<br>The cell size analysis for the analysis of hypertrophy in sections of the infarcted hearts from the different mutants were analyzed using ImageJ.

For manuscripts utilizing custom algorithms or software that are central to the research but not yet described in published literature, software must be made available to editors and reviewers. We strongly encourage code deposition in a community repository (e.g. GitHub). See the Nature Portfolio guidelines for submitting code & software for further information.

Data

Policy information about availability of data

All manuscripts must include a data availability statement. This statement should provide the following information, where applicable:

- Accession codes, unique identifiers, or web links for publicly available datasets
- A description of any restrictions on data availability
- For clinical datasets or third party data, please ensure that the statement adheres to our policy

The RNA-sequencing data of WT and Swhtr Null cardiac slices following hypoxia and recovery are deposited to GEO and can be downloaded under the accession number GSE200380 (https://www.ncbi.nlm.nih.gov/geo/query/acc.cgi?acc=GSE200380). The NKX2-5 ChIP-sequencing data from adult heart apex tissue21 used is publicly available at GEO under the accession number GSM3518668 (https://www.ncbi.nlm.nih.gov/geo/query/acc.cgi?acc=GSM3518668). The RNA-sequencing tracks from hiPSC derived cardiomyocytes is publicly available at ENCODE32 under the accession number ENCSR379YAE (https://www.encodeproject.org/experiments/ENCSR379YAE/). The cDNA of Sweetheart RNA (Swhtr) is deposited with GenBank under ON351017 (https://www.ncbi.nlm.nih.gov/nuccore/ON351017). Source data are provided with this paper.

Research involving human participants, their data, or biological material

Policy information about studies with human participants or human data. See also policy information about sex, gender (identity/presentation), and sexual orientation and race, ethnicity and racism.

Reporting on sex and gender	The human data in this manuscript are the RNA-seq tracks and the qPCR data in Figure S6B. The ENCODE derived RNA-seq data were downloaded and generated from in vitro derived cardiomyocytes from human female stem cells (RUES2). The qPCR data from in vitro derived cardiomyocytes from a commercially available male iPSC cell line (WTSIi081-A).
Reporting on race, ethnicity, or other socially relevant groupings	The ENCODE data for RUES2 do not specify these parameters. The human WTSIi081-A iPSC cell line was available by purchase, but the Biobank specifies their origin as white, British male.
Population characteristics	No population analysis are reported in this manuscript.
Recruitment	No humans were recruited for this study.
Ethics oversight	The ethics board of the Goethe University, Faculty of Medicine, approved the use of the iPSC line for this study under the approval number 2023-1503-Anfrage.

Note that full information on the approval of the study protocol must also be provided in the manuscript.

Field-specific reporting

Please select the one below that is the best fit for your research. If you are not sure, read the appropriate sections before making your selection.

☒ Life sciences ☐ Behavioural & social sciences ☐ Ecological, evolutionary & environmental sciences

For a reference copy of the document with all sections, see nature.com/documents/nr-reporting-summary-flat.pdf

Life sciences study design

All studies must disclose on these points even when the disclosure is negative.

Sample size	The sample size for the Murine Left Anterior Descending (LAD) Coronary Artery experiments was selected on the basis of number of animals that can be handeled in one experiment. The sample sizes are given for each experiment. The experiments with several mice of each genotype were repeated once with an indpendent bred cohort of mice and for the final analysis both cohorts were combined. No initial statistical determination of sample size was conducted, but community standards of an n higher than 4 was greatly succeeded by the use of all available male mice received from the breedings. Mice used were from different litters.<br>For the determination of hypertrophy heart sections from 3 individuals of each genotype were used and combined for the final automated image analysis using ImageJ without subsequent manual adjustments of the researchers.<br>For the smFISH analysis at least 50 cells were counted for each genotype as mandatory by community standards.
Data exclusions	No data were excluded.
Replication	The Murine Left Anterior Descending (LAD) Coronary Artery Ligation experiments was repeated with 2 cohorts of mice from different litters each independently, with appx half a year in between. For the final data both groups were combined and no difference was observed between these independent cohorts, confirming the reproducibility.
Randomization	The surgeon for the Murine Left Anterior Descending (LAD) Coronary Artery experiments did not know the genotype at the time of the surgery and the animals of the respective genotype were handed for surgery randomly. All mice from the pool were derived form differnet litter and were of the same age range. The obtained data from the ultrasound for this manuscript was analyzed by another person than from the person who performed the surgery.

Blinding	The surgeon for the Murine Left Anterior Descending (LAD) Coronary Artery experiments did not know the genotype at the time of the surgery. The data for this manuscript was analyzed by another person than from the person who performed the surgery. The counting for the smFISH analysis was blinded for the analyzing researcher.

Reporting for specific materials, systems and methods

We require information from authors about some types of materials, experimental systems and methods used in many studies. Here, indicate whether each material, system or method listed is relevant to your study. If you are not sure if a list item applies to your research, read the appropriate section before selecting a response.

Materials & experimental systems

n/a	Involved in the study	
☐	☒	Antibodies
☐	☒	Eukaryotic cell lines
☒	☐	Palaeontology and archaeology
☐	☒	Animals and other organisms
☒	☐	Clinical data
☒	☐	Dual use research of concern
☒	☐	Plants

Methods

n/a	Involved in the study	
☒	☐	ChIP-seq
☒	☐	Flow cytometry
☒	☐	MRI-based neuroimaging

Antibodies

Antibodies used	NKX2-5 antibody (R&D Systems #AF2444 Lot UQW0120031) for NKX2-5 precipitation, NKX2.5 Antibody (Cell signaling #5444 Lot 4) for detection on western blot. As IgG negative control for Immunoprecipitation normal goat IgG negative control (R&D Systems #AB-108-C Lot ES4521111) was used.
Validation	All antibodies were tested validated and tested prior the experiment. In total 4 different NKX2-5 antibodies were initially selected and the 2 reported antibodies that were validated for either pull down or western blot detection were used and are described. The validation was conducted by using 5 µg of each antibody for NKX2-5 pulldown and detecting it with every antibody by Western Blotting together with Input samples. The combination with the best signal to noise ratio was chosen for subsequent experiments. Concentrations are provided in the Methods section and the uncropped blot can be found in the source data set.

Eukaryotic cell lines

Policy information about cell lines and Sex and Gender in Research

Cell line source(s)	The F1G4 mESC cells were obtained from A. Nagy lab and can not be purchased from a company. The HL-1 cell line (Sigma-Aldrich #SCC065) were a gift from Prof. Ralf Gilsbach. The WTSIi081-A cells (EBiSC #66540196) were purchased from EBISC2 by Prof. Jaya Krishnan. The human iPSC were a commercially available cell line that does not require ethics ovesight on our side.
Authentication	The cell line was teste for expression of cardiomyocyte specific genes for verification prior to use for the experiments.
Mycoplasma contamination	The cell lines used in this study were tested for mycoplasms and were tested negative prior to usage.
Commonly misidentified lines (See ICLAC register)	None of these lines were used in this study.

Animals and other research organisms

Policy information about studies involving animals; ARRIVE guidelines recommended for reporting animal research, and Sex and Gender in Research

Laboratory animals	Mice were housed at an artificial 12h/12h light-dark cycle. Fortified complete mouse feed (Ssniff #V1534-000) and water available ad libitum. The humidity and ambient temperature in the housing facility were controlled daily at a temperature of around 21 °C and a relative humidity of around 49%. All experimental procedures and maintenance were carried out in accordance with the animal welfare act and agreement of the responsible authorities (specifics given in the respective sections). Animals were monitored daily to ensure well-being. Only male mice were used for AMI experiments and hence subsequent experiments. Breeding of animals was conducted for strain maintenance using homozygous x homozygous breeding as they showed no phenotype and thus mouse numbers due to absence of "wrong-genotype" littermates could be reduced. Only when animals were required for experiments, the breeding was increased to keep animal numbers low. Mice were euthanised by cervical dislocation for collection of tissue.
Wild animals	No wild animals were used in this study
Reporting on sex	The animals used for the Murine Left Anterior Descending (LAD) Coronary Artery Ligation experiments were only of the male sex to

Reporting on sex	minimize variations due to the sex. For the heart slice culture and the ex vivo cultication of cardiomyocytes samples from both sexes were used.
Field-collected samples	No field-samples were collected for this study.
Ethics oversight	All animal experiments were approved by the local authorities. The mutants were generated under the license number G0349/13 granted by the Landesamt für Gesundheit und Soziales Berlin (LAGeSo), Berlin. The animal surgery experiments have been approved by the Government Veterinary Office (Service de la Consommation et des Affaires Vétérinaires - SCAV, Epalinges, Switzerland; License number: VD2027.3, VD3275) and were carried out in accordance with the institutional guidelines of the University of Lausanne, Lausanne, Switzerland, as well as Swiss laws concerning animal protection. The animal experiments were reported according to the ARRIVE guidelines.

Note that full information on the approval of the study protocol must also be provided in the manuscript.
